# Comparative effectiveness of sodium-glucose cotransporter-2 inhibitors for new-onset gastric cancer and gastric diseases in patients with type 2 diabetes mellitus: a population-based cohort study

**DOI:** 10.1007/s10120-024-01512-7

**Published:** 2024-06-10

**Authors:** Oscar Hou In Chou, Vinod Kumar Chauhan, Cheuk To Skylar Chung, Lei Lu, Teddy Tai Loy Lee, Zita Man Wai Ng, Karin Kai Wing Wang, Sharen Lee, Haipeng Liu, Ronald Ting Kai Pang, Apichat Kaewdech, Bernard Man Yung Cheung, Gary Tse, Jiandong Zhou

**Affiliations:** 1 Division of Clinical Pharmacology, Department of Medicine, School of Clinical Medicine, Li Ka Shing Faculty of Medicine, University of Hong Kong, Hong Kong, China; 2 Institute of Biomedical Engineering, Department of Engendering Science, University of Oxford, Oxford, UK; 3 Diabetes Research Unit, Cardiovascular Analytics Group, PowerHealth Research Institute, Hong Kong, China; 4 Research Centre for Intelligent Healthcare, Coventry University, Coventry, UK; 5 School of Nursing and Health Studies, Hong Kong Metropolitan University, Hong Kong, China; 6 Division of Internal Medicine, Faculty of Medicine, Prince of Songkla University, Hat Yai, Thailand; 7 Tianjin Key Laboratory of Ionic-Molecular Function of Cardiovascular Disease, Department of Cardiology, Tianjin Institute of Cardiology, Second Hospital of Tianjin Medical University, Tianjin, 300211 China; 8 Kent and Medway Medical School, Canterbury Christ Church University and University of Kent, Canterbury, UK; 9 Department of Family Medicine and Primary Care, Li Ka Shing Faculty of Medicine, The University of Hong Kong, Hong Kong, China; 10 School of Public Health, Li Ka Shing, Faculty of Medicine, The University of Hong Kong, Hong Kong, China; 11 Department of Pharmacology and Pharmacy, The University of Hong Kong, Hong Kong, China

**Keywords:** Sodium-glucose cotransporter 2 inhibitors (SGLT2I), Dipeptidyl peptidase-4 inhibitors (DPP4I), Glucagon-like peptide-1 receptor agonist (GLP1a), Peptic ulcer, Gastric cancer, Gastritis

## Abstract

**Objective:**

To compare the risks of gastric cancer and other gastric diseases in patients with type-2 diabetes mellitus (T2DM) exposed to sodium-glucose cotransporter 2 inhibitors (SGLT2I), dipeptidyl peptidase-4 inhibitors (DPP4I) or glucagon-like peptide-1 receptor agonists (GLP1a).

**Design:**

This was a population-based cohort study of prospectively collected data on patients with T2DM prescribed SGLT2I, DPP4I or GLP1a between January 1st 2015 and December 31st 2020 from Hong Kong. The outcomes were new-onset gastric cancer, peptic ulcer (PU), acute gastritis, non-acute gastritis, and gastroesophageal reflux disease (GERD). Propensity score matching (1:1) using the nearest neighbour search was performed, and multivariable Cox regression was applied. A three-arm comparison between SGLT2I, DPP4I and GLP1a was conducted using propensity scores with inverse probability of treatment weighting.

**Results:**

A total of 62,858 patients (median age: 62.2 years old [SD: 12.8]; 55.93% males; SGLT2I: n = 23,442; DPP4I: n = 39,416) were included. In the matched cohort, the incidence of gastric cancer was lower in SGLT2I (Incidence rate per 1000 person-year, IR: 0.32; 95% confidence interval, CI 0.23–0.43) than in DPP4I (IR per 1000 person-year: 1.22; CI 1.03–1.42) users. Multivariable Cox regression found that SGLT2I use was associated with lower risks of gastric cancer (HR 0.30; 95% CI 0.19–0.48), PU, acute gastritis, non-acute gastritis, and GERD (p < 0.05) compared to DPP4I use. In the three-arm analysis, GLP1a use was associated with higher risks of gastric cancer and GERD compared to SGLT2I use.

**Conclusions:**

The use of SGLT2I was associated with lower risks of new-onset gastric cancer, PU, acute gastritis, non-acute gastritis, and GERD after matching and adjustments compared to DPP4I use. SGLT2I use was associated with lower risks of GERD and gastric cancer compared to GLP1a use.

**Graphical abstract:**

**Figure:**
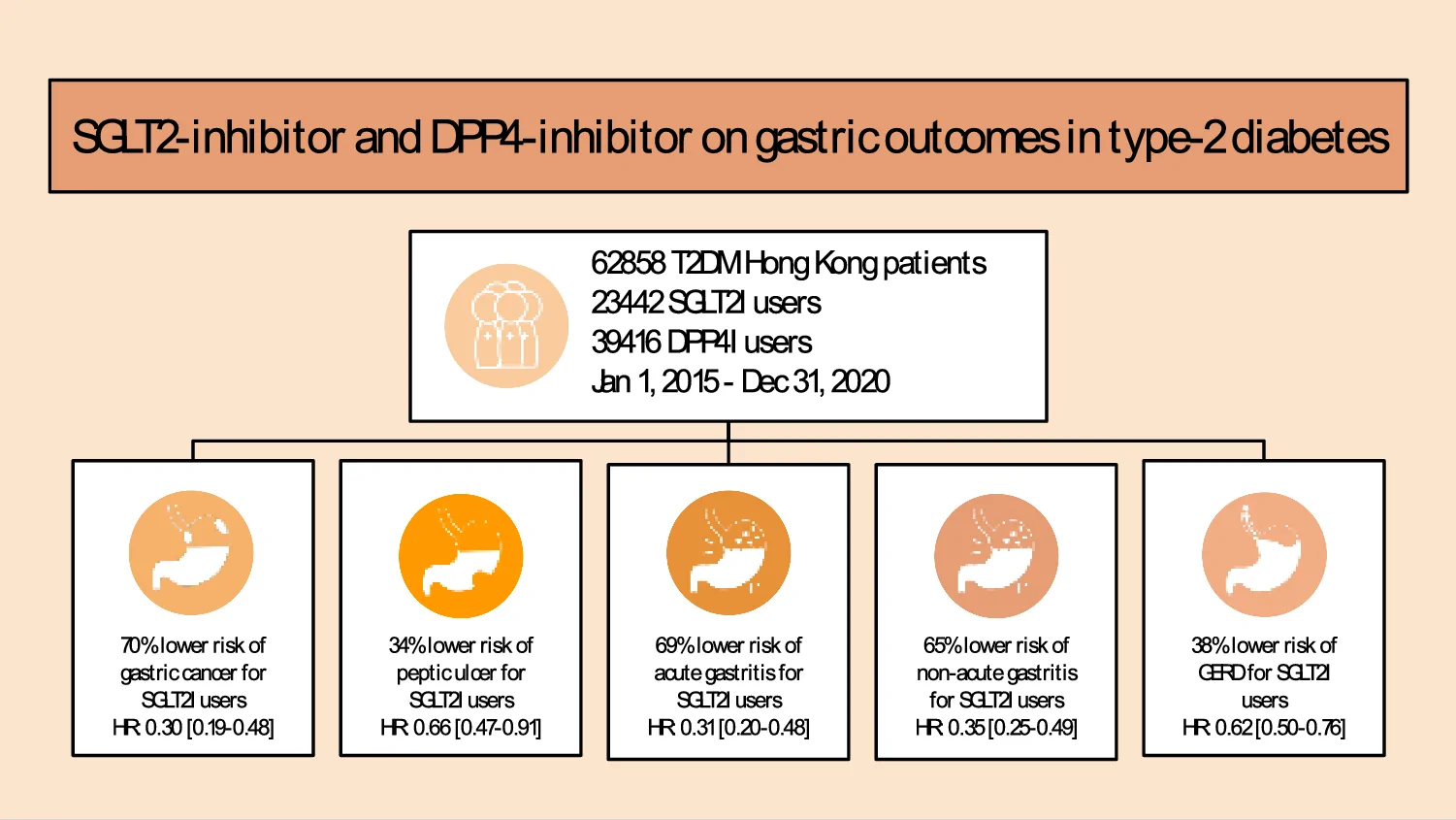

**Supplementary Information:**

The online version contains supplementary material available at https://doi.org/10.1007/s10120-024-01512-7.

## Introduction

The rising incidence of type 2 diabetes mellitus (T2DM) is a major global concern, imposing a significant burden on healthcare systems worldwide. T2DM is known to cause multiple complications, including upper gastrointestinal (GI) tract complications. Gastric cancer remained one of the most common causes of cancer-related mortality in China, with an estimated 5-year overall survival rate of 40.7% among 241 prospective studies conducted from 2000 to 2022 based on a nationwide systematic review [1]. A systematic review of cohort studies has revealed a 14% higher risk of gastric cancer in individuals with T2DM [2]. Gastric cancer is more prevalent in Asia, with about half of the total gastric cancer cases found in the region, especially in China [3, 4].

Alongside gastric cancer, there is growing evidence regarding the role of diabetes in various types of gastric diseases. Patients with diabetes were found to be at higher risk for developing peptic ulcers [5]. Therefore, the evidence raised the question of whether anti-diabetic agents had protective effects against gastric diseases. Experimental studies have found that metformin produced ulcer healing effects comparable to that of omeprazole, effects attributed to its anti-inflammatory actions [6]. These findings offered a potential explanation for the lower incidence of gastric cancer among people with diabetes who undergo eradication of *Helicobacter pylori* (*H. pylori*) [7].

However, the current literature surrounding novel anti-diabetic agents such as sodium-glucose cotransporter 2 inhibitors (SGLT2I) and dipeptidyl peptidase 4 inhibitors (DPP4I) on various gastric diseases remain controversial. Recent studies suggested that SGLT2I may offer potential GI protective effects. A meta-analysis of randomized controlled trials demonstrated an association between canagliflozin and a decreased risk of GI cancers, but no significant association was found with other SGLT2Is [8]. Possible biological mechanisms of SGLT2I on reduction of cancer risk include reduction of glucose uptake of cancer cells, thereby increasing cell necrosis and reducing tumour growth [9], increased insulin sensitivity, and reduced chronic inflammation [10–12]. DPP4I and GLP1a may exert anti-cancer effects on gastric cancer via similar mechanisms [13, 14].

As of now, there is limited clinical evidence surrounding the association of the use of novel second-line anti-diabetic drugs (SGLT2I, DPP4I and glucagon-like peptide-1 receptor agonist (GLP1a) on different types of gastric diseases. Hence, the present study aims to explore the role of SGLT2I, DPP4I and GLP1a on new-onset gastric cancer and gastric diseases in a cohort of T2DM patients from Hong Kong.

## Methods

### Study population

This was a retrospective population-based study based on the Clinical Data Analysis and Reporting System (CDARS). CDARS encompasses medical records for over 90% of Hong Kong's population, cataloguing a wealth of information including disease diagnoses, laboratory results, past comorbidities, clinical characteristics, and medication prescriptions [15]. CDARS is managed by the Hospital Authority, a statutory body overseeing all 43 public hospitals and 123 outpatient clinics across Hong Kong’s seven geographic regions, ensuring a population-based sample that is representative of the city [16]. CDARS has been employed in numerous populated-based studies [17–19]. This study was approved by the Institutional Review Board of the University of Hong Kong/Hospital Authority Hong Kong West Cluster (HKU/HA HKWC IRB) (UW-20-250) and complied with the Declaration of Helsinki. This study employed a new-user study design, in which T2DM patients who were newly prescribed either SGLT2I or only DPP4I in centres under the Hong Kong Hospital Authority, between 1st January 2015 and 31st December 2020 (Supplementary Fig. 7). The GLP1a cohort comprised of patients newly prescribed GLP1a during the same period of time and was included for 3-arm comparison.

### Input variables

The main exposure variable was SGLT2I or DPP4I uses. The following variables were extracted: gender, age of initial use of SGLT2I or DPP4I, clinical, laboratory, and medication data. Prior comorbidities were extracted in accordance with the *International Classification of Diseases Ninth Edition* (ICD-9) codes (Supplementary Table 1). Patient with prior identified *H. pylori* infection were defined by either ICD-9: 041.86, Microbiology results (MIS data), or prior history of *H. pylori* eradication therapy with concomitant use of proton pump inhibitor (PPI) and antibiotics according to previous study [20]. The diabetes duration was calculated by examining the earliest date amongst the first date of (1) diagnosis using ICD-9; (2) Haemoglobin A1c (HbA1c) ≥ 6.5%; (3) Fasting glucose ≥ 7.0 mmol/l or Random glucose ≥ 11.1 mmol/l [21]; (4) Using insulin and anti-diabetic medications apart from SGLT2I, DPP4I, and GLP1a. The Charlson's standard comorbidity index was calculated [22]. Patients with hepatitis C virus (HCV) infection were defined by both the ICD-9 code and HCV RNA positive status. Patients with hepatitis B virus (HBV) infection were defined by both the ICD 9 code and positive hepatitis B surface antigen (HBsAg) positive status.

The GI medications, cardiovascular medications, anti-diabetic agents, and the antibiotics were extracted from the database. The duration and frequency of SGLT2I and DPP4I usage were extracted. Moreover, the baseline laboratory examinations, including the complete blood count, lipid and glucose profiles, as well as liver and renal biochemical tests were extracted. The estimated glomerular filtration rate (eGFR) was calculated using the abbreviated modification of diet in renal disease (MDRD) formula [23]. The variability measure for the lipid and glucose profiles were also calculated to reflect the extent of diabetes control (Supplementary Table 2). Furthermore, the time-weighted lipid and glucose profiles after drug initiation were also calculated by the products of the sums of two consecutive measurements and the time interval, then divided by the total time interval, as suggested previously [24].

### Outcome of the study

The primary outcome of this study was the development of gastric cancer, peptic ulcer, acute gastritis, non-acute gastritis, and gastroesophageal reflux disease (GERD) upon the index date of the drug use (Supplementary Table 1). The secondary outcome was all-cause mortality. Mortality data were obtained from the Hong Kong Death Registry, a population-based official government registry with the registered death records of all Hong Kong citizens linked to CDARS. Mortality was recorded using the *International Classification of Diseases Tenth Edition* (ICD-10) coding. The endpoint date of interest for eligible patients was the event presentation date. The endpoint for those without primary outcome presentation was the mortality date or the endpoint of the study (31st December 2020).

### Statistical analysis

Descriptive statistics are used to summarize baseline clinical and biochemical characteristics of patients with SGLT2I and DPP4I use. For baseline clinical characteristics, continuous variables were presented as mean (95% confidence interval [95% CI]/standard deviation [25]) and the categorical variables were presented as total numbers (percentage). Propensity score matching with 1:1 ratio for SGLT2I use versus DPP4I use based on demographics, Charlson comorbidity index, non-SGLT2I/DPP4I medications, prior comorbidities, biomarkers and duration from T2DM diagnosis initial drug exposure were performed using the nearest neighbour search strategy with a calliper of 0.1. Propensity score matching was performed using Stata software (Version 16.0).

Baseline characteristics between patients with SGLT2I and DPP4I use before and after matching were compared with absolute standardized mean difference (SMD), with SMD < 0.10 regarded as well-balanced between the two groups. The cumulative incidence curves for the primary outcomes and secondary outcomes were constructed and compared for the risk using log-rank tests. Proportional Cox regression models were used to identify significant risk predictors of adverse study outcomes. The log–log plot was used to verify the proportionality assumption for the proportional Cox regression models. Subgroup analysis was conducted to confirm the association amongst patients with different clinical important predictors. Cause-specific and sub-distribution hazard models were conducted to consider possible competing risks. Multiple propensity adjustment approaches were used, including propensity score stratification [26], propensity score matching with inverse probability of treatment weighting [27] and stable inverse probability weighting [28].

The three arm comparison results involving GLP1a using stabilized inverse probability of treatment weighting (IPTW) were conducted to provide further information regarding the gastric effects of the novel second-line anti-diabetic medications**.** Multiple sensitivity analysis were conducted to show the robustness of the associations. Furthermore, patients with chronic kidney disease (CKD) stage 4/5 (eGFR < 30), peritoneal dialysis or haemodialysis who may be contraindicated with SGLT2I were excluded in the analysis. The analysis results with consideration of one-year lag time effects was also conducted. The as-treat approach was conducted, which patient were censored at treatment discontinuation or switching of the comparison medications. The negative control outcome was suggested to detect the residual bias and confounding factors due to unobserved confounders. We used the venous thromboembolism as the negative control in the falsification analysis (Supplementary Table 1), such that the observed significant association in the falsification analysis should be attributed to bias. The hazard ratio (HR), 95% CI and P value were reported. Statistical significance was defined as p value < 0.05. All statistical analyses were performed with RStudio (Version: 1.1.456) and Python (Version: 3.6).

## Results

### Basic characteristics

**Fig. 1:**
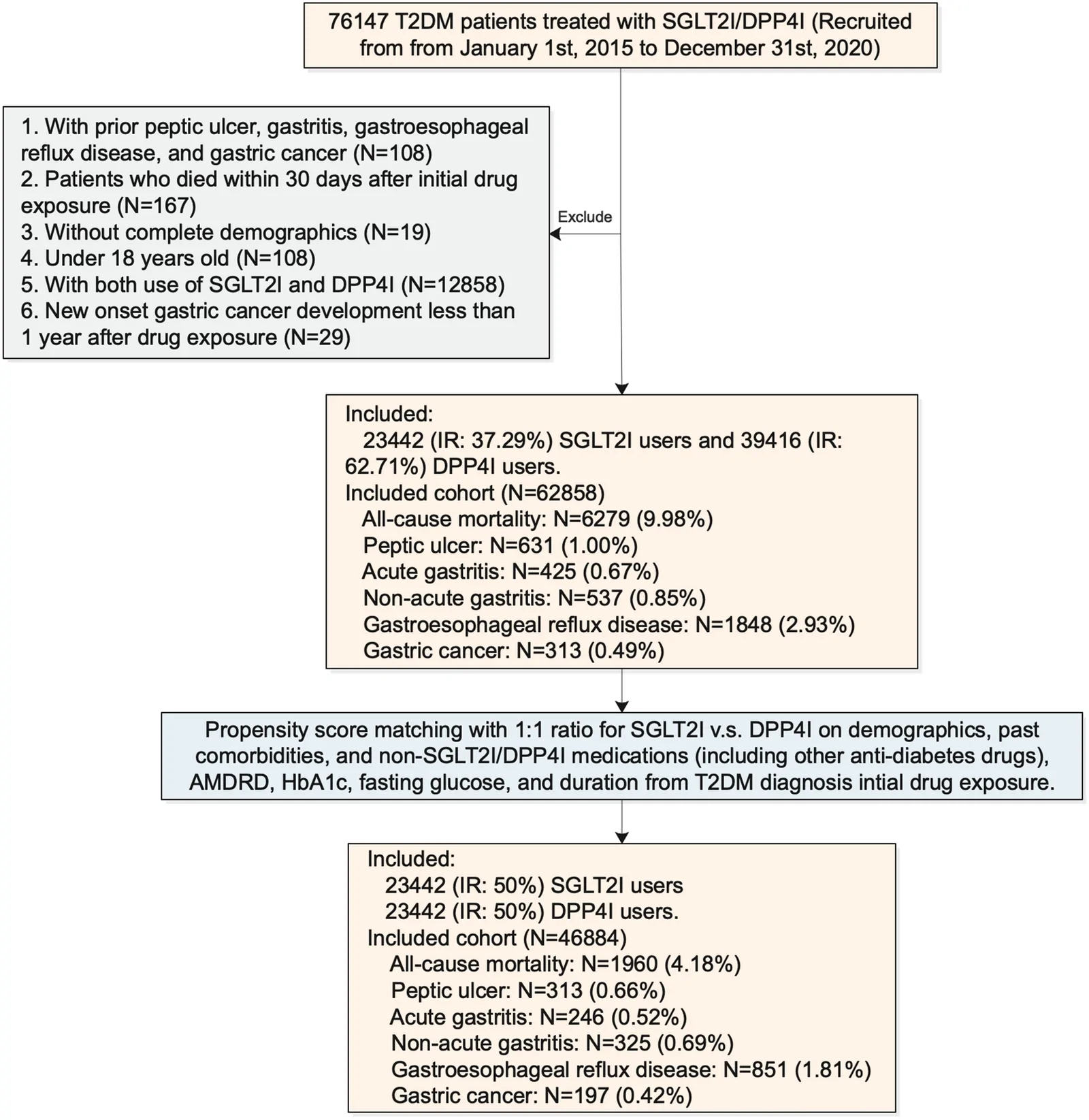
Procedures of data processing for the study cohort. *SGLT2I* Sodium-glucose cotransporter-2 inhibitors, *DPP4I* Dipeptidyl peptidase-4 inhibitors. *MDRD* modification of diet in renal disease

In this territory-wide cohort study of 76,147 patients with T2DM newly treated with SGLT2I/DPP4I between 1st January 2015 and 31st December 2020 in Hong Kong, patients were followed up until 31st December 2020 or until their deaths (Fig. 1). The following patient groups were excluded: those who (1) died within 30 days after initial drug exposure (N = 167); (2) without complete demographics (N = 19); (3) under 18 years old (N = 108); (4) with prior peptic ulcer, gastritis, GERD, gastric cancer (N = 108); (5) exposed to both DPP4I and SGLT2I prescription at any time point (N = 12,858) and (6) new onset gastric cancer development less than 1 year after drug exposure (N = 29).

After exclusion, this study included a total of 62,858 patients with T2DM (mean age: 62.2 years old [SD: 12.8]; 55.93% males), of whom 23,442 patients (35.33%) used SGLT2Is, and 39,416 patients (64.67%) used DPP4Is (Table 1). Before matching, the SGLT2I users were younger, with less comorbidities, more patients were using anti-diabetic drugs thiazolidinedione and GLP1a, and have a higher eGFR compared to DPP4I users. The distribution from T2DM diagnosis date, and the duration from the medication initiation to clinical outcomes for SGLT2I, DPP4I, and GLP1a users are shown in Supplementary Fig. 1A and 1B respectively. The drug initiation date of the SGLT2I, DPP4I and GLP1a is shown in Supplementary Fig. 1C.

**Table 1:** Baseline and clinical characteristics of patients with SGLT2I v.s. DPP4I use before and after propensity score matching (1:1)

Characteristics	Before matching				After matching			
	All (N = 62,858) Mean(SD); N or Count(%)	SGLT2I users (N = 23,442) Mean(SD); N or Count(%)	DPP4I users (N = 39,416) Mean(SD); N or Count(%)	SMD^#^	All (N = 46,884) Mean(SD); N or Count(%)	SGLT2I users (N = 23,442) Mean(SD); N or Count(%)	DPP4I users (N = 23,442) Mean(SD); N or Count(%)	SMD^#^
Demographics								
Male gender	35,160 (55.93)	13,979 (59.63)	21,181 (53.73)	0.12	27,992 (59.70)	13,979 (59.63)	14,013 (59.77)	< 0.01
Female gender	27,698 (44.06)	9463 (40.36)	18,235 (46.26)	0.12	18,892 (40.29)	9463 (40.36)	9429 (40.22)	< 0.01
Baseline age, years	62.2 (12.8)	57.7 (11.3)	64.9 (12.9)	0.59	58.0 (11.1)	57.7 (11.3)	58.4 (10.9)	0.06
Past comorbidities								
Charlson comorbidity index	2.0 (1.5)	1.6 (1.3)	2.3 (1.6	0.52	1.6 (1.2)	1.56 (1.25)	1.58 (1.23)	0.02
Duration from earliest diabetes mellitus diagnosis to index date, day	509.9 (1214.8)	519.6 (1332.2)	504.1 (1139.2)	0.01	507.8 (1254.5)	519.6 (1332.2)	495.9 (1171.4)	0.02
Hypertension	14,514 (23.09)	5255 (22.41)	9259 (23.49)	0.03	10,149 (21.64)	5255 (22.41)	4894 (20.87)	0.04
Heart failure	2080 (3.30)	634 (2.70)	1446 (3.66)	0.05	1242 (2.64)	634 (2.70)	608 (2.59)	0.01
Ischemic heart disease	6381 (10.15)	2904 (12.38)	3477 (8.82)	0.12	5575 (11.89)	2904 (12.38)	2671 (11.39)	0.03
Atrial fibrillation	1604 (2.55)	526 (2.24)	1078 (2.73)	0.03	1045 (2.22)	526 (2.24)	519 (2.21)	< 0.01
Acute myocardial infarction	1775 (2.82)	798 (3.40)	977 (2.47)	0.05	1565 (3.33)	798 (3.40)	767 (3.27)	0.01
Peripheral vascular disease	588 (0.93)	193 (0.82)	395 (1.00)	0.02	384 (0.81)	193 (0.82)	191 (0.81)	< 0.01
Renal diseases	1140 (1.81)	151 (0.64)	989 (2.50)	0.15	301 (0.64)	151 (0.64)	150 (0.63)	< 0.01
Stroke/transient ischemic attack	1989 (3.16)	615 (2.62)	1374 (3.48)	0.05	1221 (2.60)	615 (2.62)	606 (2.58)	< 0.01
Gastrointestinal bleeding	1434 (2.28)	414 (1.76)	1020 (2.58)	0.06	815 (1.73)	414 (1.76)	401 (1.71)	< 0.01
Identified *H. pylori* infection	3597 (5.72)	1231 (5.25)	2366 (6.00)	0.03	2388 (5.09)	1231 (5.25)	1157 (4.93)	0.01
Autoimmune disease	653 (1.03)	246 (1.04)	407 (1.03)	< 0.01	480 (1.02)	246 (1.04)	234 (0.99)	0.01
Cancer	1604 (2.55)	448 (1.91)	1156 (2.93)	0.07	894 (1.90)	448 (1.91)	446 (1.90)	< 0.01
Chronic liver disease and cirrhosis	1312 (2.08)	633 (2.70)	679 (1.72)	0.07	1245 (2.65)	633 (2.70)	612 (2.61)	0.01
HBV infection	2640 (4.19)	1232 (5.25)	1408 (3.57)	0.08	2365 (5.04)	1232 (5.25)	1133 (4.83)	0.02
HCV infection	191 (0.30)	70 (0.29)	121 (0.30)	< 0.01	140 (0.29)	70 (0.29)	70 (0.29)	< 0.01
Other liver diseases	711 (1.13)	222 (0.94)	489 (1.24)	0.03	441 (0.94)	222 (0.94)	219 (0.93)	< 0.01
Diabetic retinopathy	4316 (6.86)	1626 (6.93)	2690 (6.82)	< 0.01	3038 (6.47)	1626 (6.93)	1412 (6.02)	0.04
Medication prescriptions								
Metformin	56,412 (89.74)	21,884 (93.35)	34,528 (87.59)	0.2	43,866 (93.56)	21,884 (93.35)	21,982 (93.77)	0.02
Sulphonylurea	48,382 (76.97)	16,902 (72.10)	31,480 (79.86)	0.18	34,646 (73.89)	16,902 (72.10)	17,744 (75.69)	0.08
Insulin	31,798 (50.58)	11,950 (50.97)	19,848 (50.35)	0.01	24,030 (51.25)	11,950 (50.97)	12,080 (51.53)	0.01
Acarbose	1676 (2.66)	890 (3.79)	786 (1.99)	0.11	1581 (3.37)	840 (3.58)	741 (3.16)	0.02
Thiozolidinedone	12,537 (19.94)	6377 (27.20)	6160 (15.62)	0.29	12,007 (25.61)	6377 (27.20)	5630 (24.01)	0.07
Glucagon-like peptide-1 receptor agonists	2017 (3.20)	1561 (6.65)	456 (1.15)	0.29	1951 (4.16)	1061 (4.52)	890 (3.79)	0.04
H2 antagonists	29,352 (46.69)	10,764 (45.91)	18,588 (47.15)	0.02	21,747 (46.38)	10,764 (45.91)	10,983 (46.85)	0.02
Proton pump inhibitors	19,309 (30.71)	7007 (29.89)	12,302 (31.21)	0.03	13,720 (29.26)	7007 (29.89)	6713 (28.63)	0.03
ACEI/ARB	20,586 (32.75)	13,846 (59.06)	6740 (17.09)	0.96	20,270 (43.23)	10,146 (43.28)	10,124 (43.18)	< 0.01
Antibiotics	41,546 (66.09)	14,883 (63.48)	26,663 (67.64)	0.09	30,127 (64.25)	14,883 (63.48)	15,244 (65.02)	0.03
Other antihypertensive drugs	2709 (4.30)	2117 (9.03)	592 (1.50)	0.34	2688 (5.73)	1517 (6.47)	1171 (4.99)	0.06
Anti-hepatitis	946 (1.50)	466 (1.98)	480 (1.21)	0.06	923 (1.96)	466 (1.98)	457 (1.94)	< 0.01
Statins and fibrates	32,843 (52.24)	17,296 (73.78)	15,547 (39.44)	0.74	32,519 (69.36)	16,696 (71.22)	15,823 (67.49)	0.08
HCV treatment	746 (1.18)	363 (1.54)	383 (0.97)	0.05	723 (1.54)	363 (1.54)	360 (1.53)	< 0.01
HBV treatment	898 (1.42)	440 (1.87)	458 (1.16)	0.06	878 (1.87)	440 (1.87)	438 (1.86)	< 0.01
Antiplatelets	11,502 (18.29)	7429 (31.69)	4073 (10.33)	0.54	32,880 (70.13)	16,440 (70.13)	16,440 (70.13)	< 0.01
Lipid-lowering drugs	22,612 (35.97)	15,358 (65.51)	7254 (18.40)	1.09	11,382 (24.27)	5829 (24.86)	5553 (23.68)	0.03
Non-steroidal anti-inflammatory drugs	11,041 (17.56)	7122 (30.38)	3919 (9.94)	0.53	20,875 (44.52)	10,358 (44.18)	10,517 (44.86)	0.01
Diuretics	11,823 (18.80)	7196 (30.69)	4627 (11.73)	0.48	10,721 (22.86)	5422 (23.12)	5299 (22.60)	0.01
Beta-blockers	9273 (14.75)	5973 (25.47)	3300 (8.37)	0.47	11,021 (23.50)	5596 (23.87)	5425 (23.14)	0.02
Calcium channel blockers	16,249 (25.85)	10,218 (43.58)	6031 (15.30)	0.65	9052 (19.30)	4573 (19.50)	4479 (19.10)	0.01
Subclinical biomarkers								
Abbreviated MDRD, mL/min/1.73 m^2	80.9 (28.5); n = 51,653	90.0 (24.2); n = 19,466	75.4 (29.5); n = 32,187	0.54	88.4 (24.8); n = 37,875	90.0 (24.2); n = 19,466	86.8 (25.3); n = 18,409	0.13
Red cell count, × 10^12/L	4.5 (0.7); n = 32,370	4.7 (0.6); n = 13,137	4.4 (0.7); n = 19,233	0.51	4.7 (0.6); n = 25,159	4.74 (0.59); n = 13,137	4.68 (0.61); n = 12,022	0.09
Liver and renal functions								
Urea, mmol/L	6.5 (3.5); n = 51,510	5.7 (2.1); n = 19,426	7.0 (4.0); n = 32,084	0.4	5.8 (2.3); n = 37,790	5.7 (2.1); n = 19,426	5.9 (2.6); n = 18,364	0.09
Creatinine, μmol/L	93.8 (74.2); n = 51,653	78.9 (29.2); n = 19,466	102.8 (90.0); n = 32,187	0.36	81.5 (41.1); n = 37,875	78.9 (29.2); n = 19,466	84.1 (50.6); n = 18,409	0.12
Bilirubin, μmol/L	11.3 (7.0); n = 40,057	11.5 (6.2); n = 16,403	11.1 (7.5); n = 23,654	0.05	11.4 (6.1); n = 31,503	11.5 (6.2); n = 16,403	11.4 (5.9); n = 15,100	0.02
Lipid/glucose profiles								
Triglyceride, mmol/L	1.7 (1.6); n = 48,474	1.8 (1.9); n = 18,581	1.7 (1.4); n = 29,893	0.08	1.8 (1.8); n = 36,107	1.83 (1.86); n = 18,581	1.83 (1.78); n = 17,526	< 0.01
Low-density lipoprotein, mmol/L	2.4 (0.8); n = 47,587	2.39 (0.81); n = 18,228	2.39 (0.8); n = 29,359	0.01	2.4 (0.8); n = 35,391	2.39 (0.81); n = 18,228	2.4 (0.79); n = 17,163	0.01
High-density lipoprotein, mmol/L	1.2 (0.3); n = 48,395	1.16 (0.31); n = 18,544	1.21 (0.34); n = 29,851	0.14	1.2 (0.3); n = 36,036	1.16 (0.31); n = 18,544	1.18 (0.31); n = 17,492	0.04
Total cholesterol, mmol/L	4.3 (1.0); n = 48,526	4.35 (1.03); n = 18,609	4.34 (0.99); n = 29,917	0.01	4.4 (1.0); n = 36,150	4.3 (1.0); n = 18,609	4.4 (1.0); n = 17,541	0.02
Baseline-Fasting glucose, mmol/L	9.0 (3.4); n = 62,858	9.2 (3.2); n = 23,442	8.9 (3.4); n = 39,416	0.09	9.2 (3.5); n = 33,684	9.2 (3.7); n = 17,561	9.1 (3.4); n = 16,123	0.04
Baseline-Hemoglobin A1C, %	8.1 (1.4); n = 62,858	8.3 (1.4); n = 23,442	8.0 (1.4); n = 39,416	0.19	8.3 (1.5); n = 37,166	8.3 (1.6); n = 19,101	8.2 (1.4); n = 18,065	0.06
Time-weighted mean of lipid/glucose profiles								
Time weighted mean of triglyceride, mmol/L	1.8 (1.0); n = 31,689	1.9 (1.0); n = 13,941	1.8 (1.0); n = 17,748	0.05	1.9 (1.1); n = 26,652	1.87 (1.02); n = 13,941	1.9 (1.09); n = 12,711	0.03
Time weighted mean of low-density lipoprotein, mmol/L	2.3 (0.6); n = 29,674	2.3 (0.57); n = 12,992	2.34 (0.59); n = 16,682	0.06	2.3 (0.6); n = 24,885	2.3 (0.57); n = 12,992	2.32 (0.58); n = 11,893	0.03
Time weighted mean of high-density lipoprotein, mmol/L	1.2 (0.2); n = 31,226	1.16 (0.19); n = 13,826	1.18 (0.21); n = 17,400	0.09	1.2 (0.2); n = 26,429	1.16 (0.19); n = 13,826	1.16 (0.19); n = 12,603	< 0.01
Time weighted mean of total cholesterol, mmol/L	4.3 (0.6); n = 31,718	4.3 (0.6); n = 13,966	4.4 (0.6); n = 17,752	0.08	4.3 (0.6); n = 26,706	4.3 (0.61); n = 13,966	4.32 (0.61); n = 12,740	0.02
Time weighted mean of fasting glucose, mmol/L	7.8 (3.0); n = 34,853	7.9 (2.9); n = 14,876	7.8 (3.0); n = 19,977	0.03	7.9 (3.1); n = 28,298	7.9 (2.9); n = 14,876	7.8 (3.2); n = 13,422	0.03
Time weighted mean of HbA1C, %	7.9 (1.4); n = 37,636	7.9 (1.4); n = 15,882	7.8 (1.4); n = 21,754	0.07	7.9 (1.4); n = 30,562	7.91 (1.39); n = 15,882	7.91 (1.36); n = 14,680	< 0.01

*SGLT2I* sodium glucose cotransporter-2 inhibitor, *DPP4I* dipeptidyl peptidase-4 inhibitor, *SD* standard deviation, *RMS* Rooted mean square, *CV* Coefficient of variation, *MDRD* modification of diet in renal disease, *ACEI* angiotensin-converting enzyme inhibitors, *ARB* angiotensin II receptor blockers, *HBV* hepatitis B virus, *HCV* hepatitis C virus, *SMD* standardized mean difference

After propensity score matching, the two treatment groups were well-balanced in terms of baseline characteristics, except for eGFR, which had a difference of 1.6 between the two groups (SMD = 0.13) (Table 1), and the proportional hazard assumption was tested (Supplementary Fig. 2). The DPP4I and SGLT2I cohorts were comparable after matching with nearest neighbour search strategy with calliper of 0.1, and the proportional hazard assumption was confirmed (Supplementary Fig. 2). In the matched cohort, 197 patients developed new onset gastric cancer. Besides, 314 patients developed peptic ulcer, 246 developed acute gastritis, 325 developed non-acute gastritis, and 851 patients developed GERD. Additionally, 1960 patients passed away during the study period. The characteristics of patients are shown in Table 1.

### Association between SGLT2I and DPP4I and gastric cancer

**Fig. 2:**
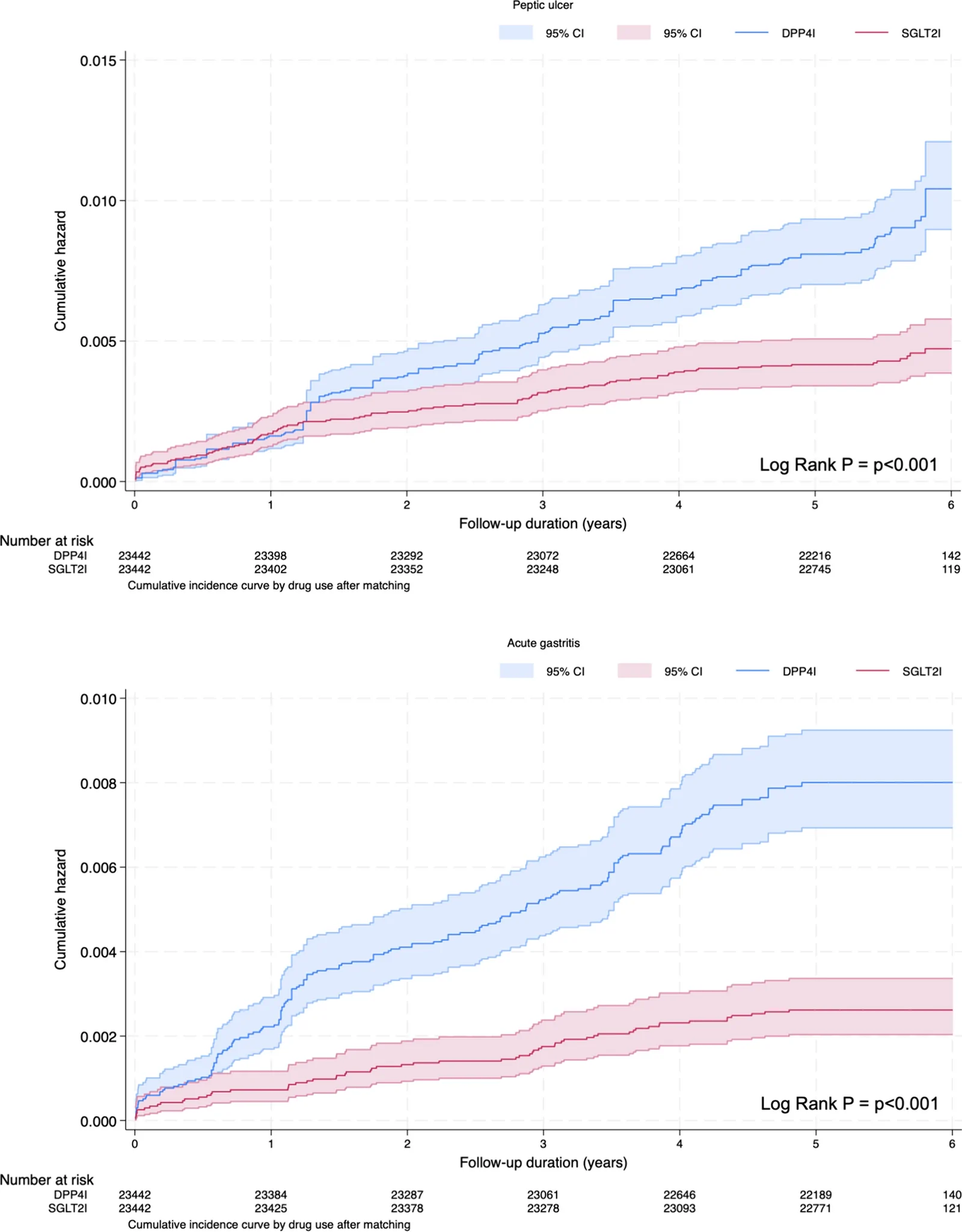

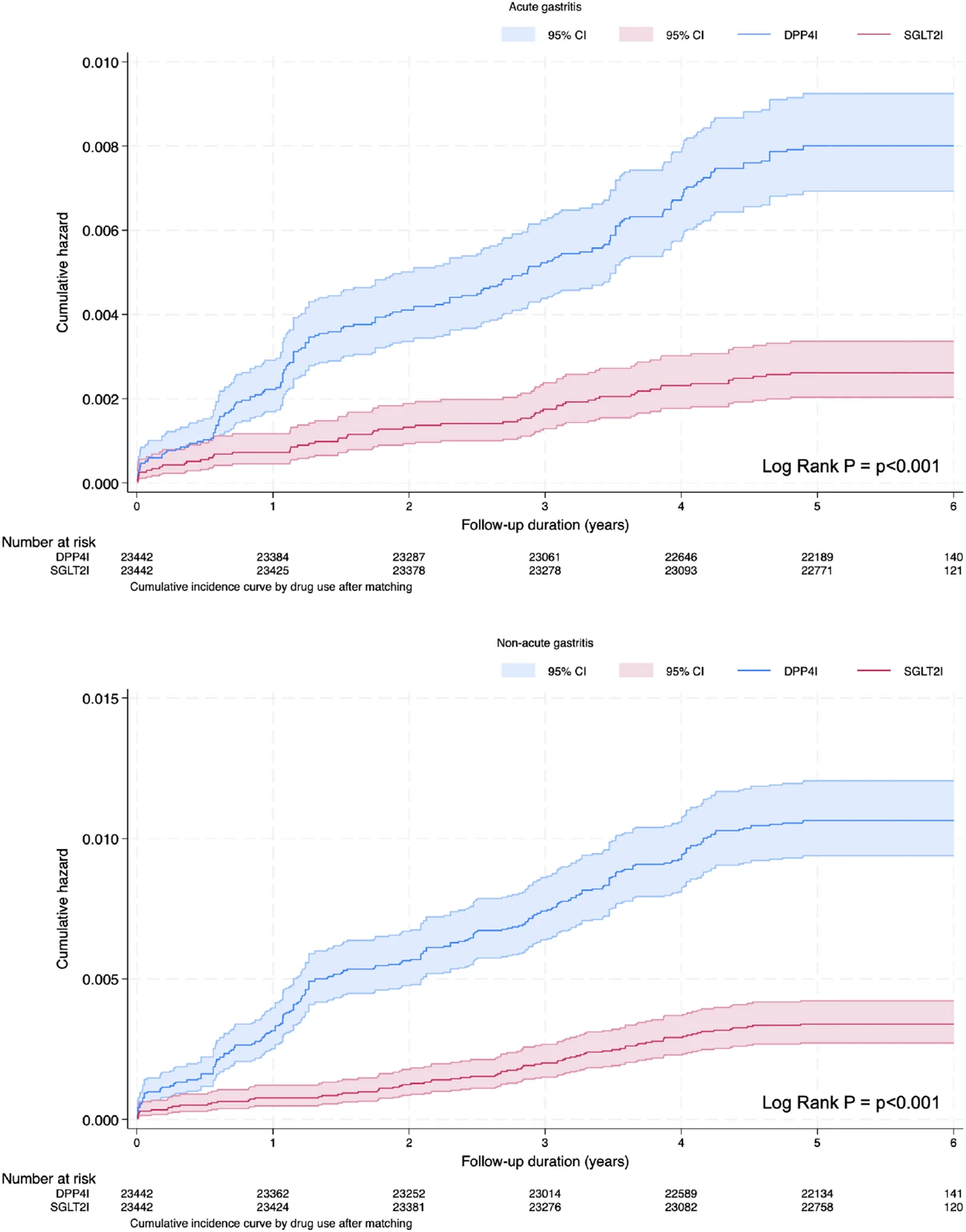

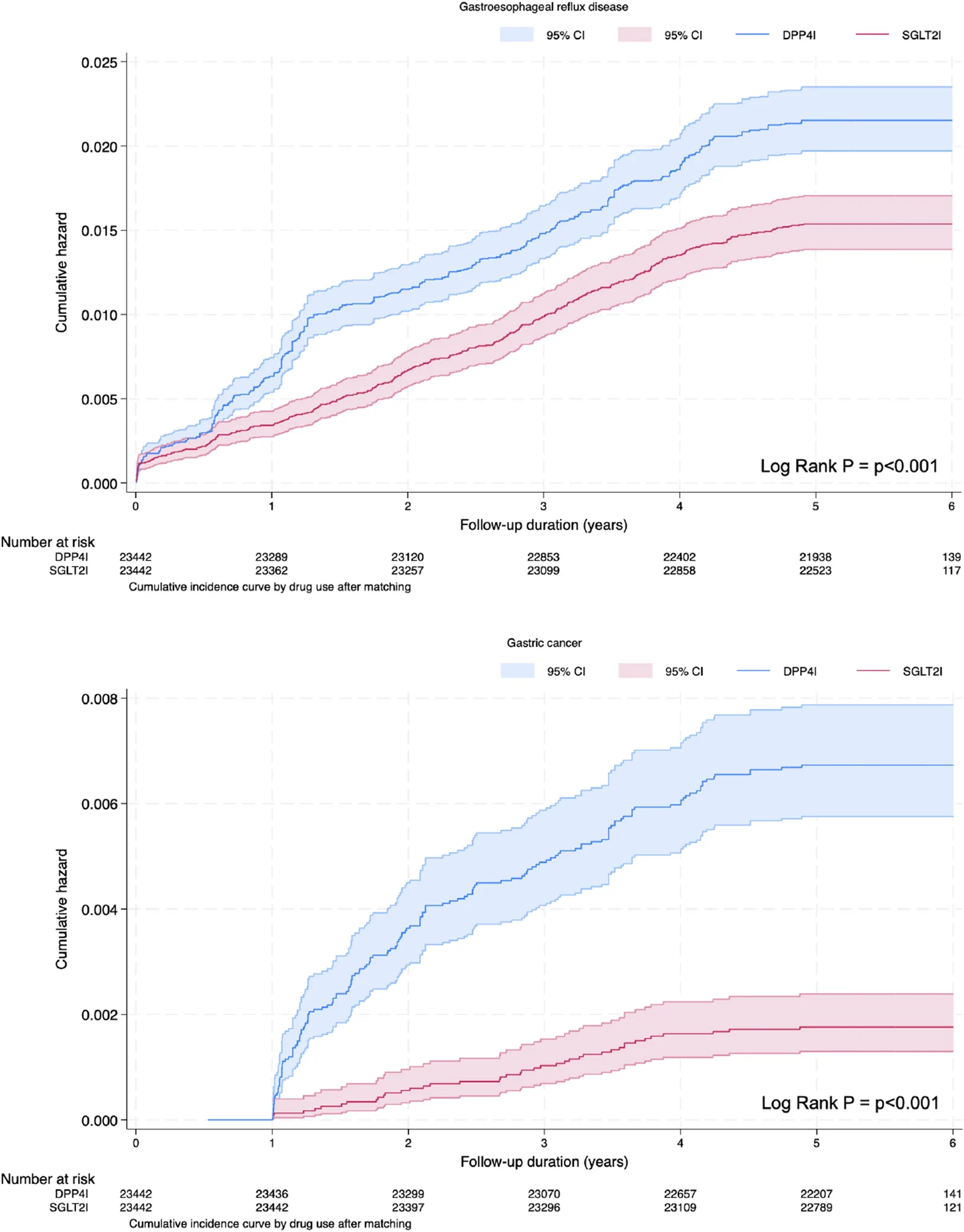

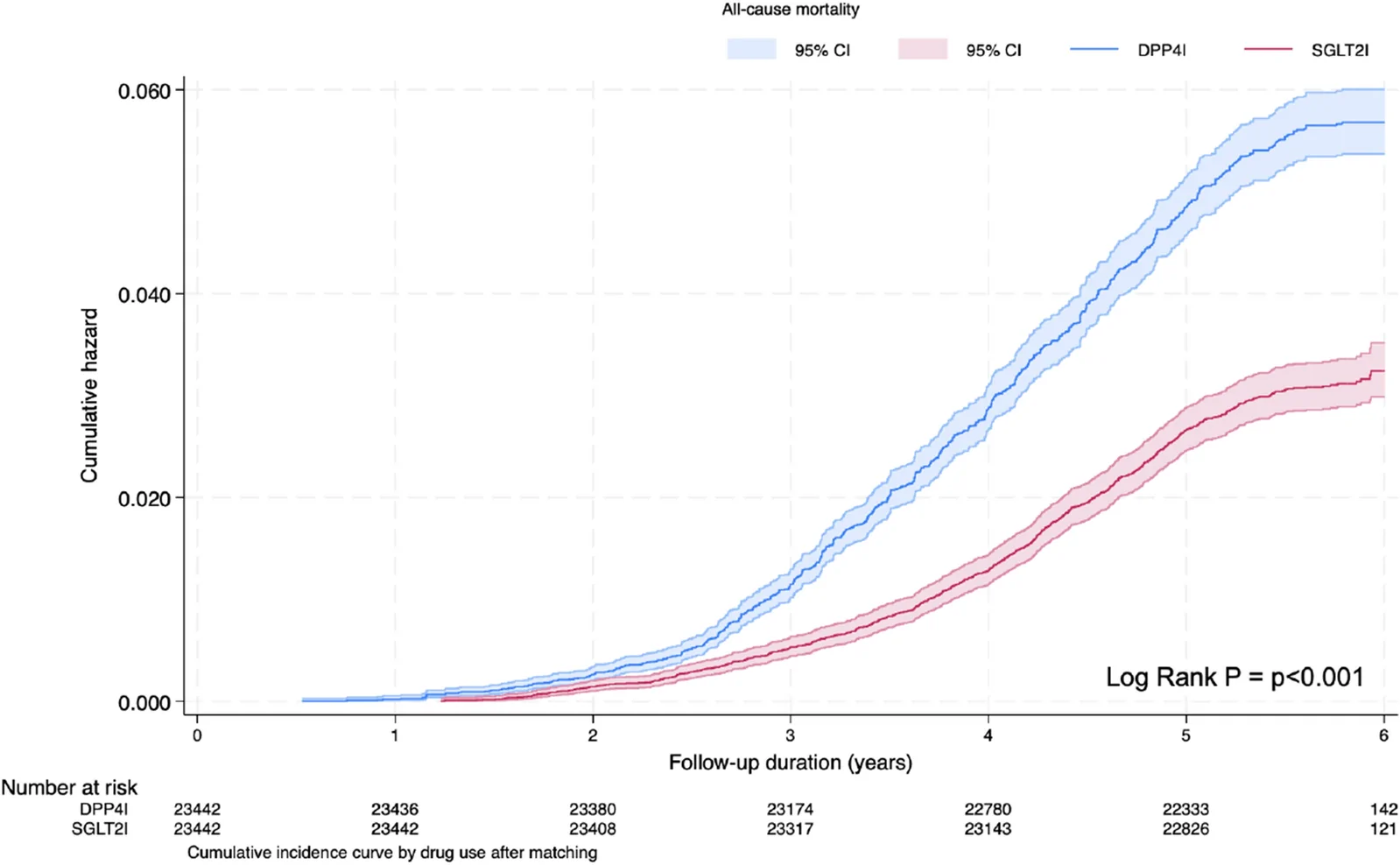
Cumulative incidence curves for new onset gastric outcomes and all-cause mortality stratified by drug exposure effects of SGLT2I and DPP4I after propensity score matching (1:1). *SGLT2I* Sodium-glucose cotransporter-2 inhibitors, *DPP4I* Dipeptidyl peptidase-4 inhibitors

In the matched cohort, there were 41 SGLT2I users and 156 DPP4I users who developed gastric cancer. After a follow-up of 257,947.7 person-year, the incidence of gastric cancer was lower amongst SGLT2I users (Incidence rate [IR] per 1000 person-year: 0.32; 95% CI 0.23–0.43) compared to DPP4I users (IR per 1000 person-year: 1.22; 95% CI 1.03–1.42) with a rate ratio of 0.26 (95% CI 0.18–0.37) (Table 2). The incidence of gastric cancer amongst DPP4I users was similar to the incidence in China amongst patients over 60 years old (1.28 per 1000 person-time) [29, 30]. SGLT2I use was associated with a lower risk of gastric cancer compared to DPP4I use after adjustment (HR: 0.30; 95% CI 0.19–0.48, p < 0.0001) regardless of the demographics, comorbidities, medication profile, renal function, inflammatory status, glycaemic tests, and the duration of T2DM (Table 2; Supplementary Fig. 3; Supplementary Table 3). This was substantiated by the cumulative incidence curves stratified by SGLT2I versus DPP4I (Fig. 2).

**Table 2:** Incidence rate (IR) per 1000 person-year and the association of primary outcomes and all-cause mortality in the SGLT2I v.s. DPP4I cohort before and after 1:1 propensity score matching

	Person-year	Number of events	IR [95% CI]	Rate ratio	Adjusted hazard ratio	P-value
Gastric cancer						
DPP4I users	128,115.3	156	1.22[1.03–1.42]	1 [Reference]	1 [Reference]	NA
SGLT2I users	129,832.4	41	0.32[0.23–0.43]	0.26[0.18–0.37]	0.30[0.19–0.48]	< 0.0001
Peptic ulcer						
DPP4I users	127,820.7	211	1.65[1.44–1.89]	1 [Reference]	1 [Reference]	NA
SGLT2I users	129,606.3	103	0.80[0.65–0.96]	0.48[0.38–0.61]	0.66[0.47–0.91];	0.0118
Acute gastritis						
DPP4I users	128,061.7	185	1.44[1.24–1.67]	1 [Reference]	1 [Reference]	NA
SGLT2I users	129,749.4	61	0.47[0.36–0.60]	0.33[0.24–0.43]	0.31[0.20–0.48];	< 0.0001
Non-acute gastritis						
DPP4I users	127,828.5	246	1.92[1.69–2.18]	1 [Reference]	1 [Reference]	NA
SGLT2I users	129,718.7	79	0.61[0.48–0.76]	0.31[0.25–0.41]	0.35[0.25–0.49];	P < 0.0001
Gastroesophageal reflux disease						
DPP4I users	127,052.7	495	3.90[3.56–4.25]	1 [Reference]	1 [Reference]	NA
SGLT2I users	128,869.4	356	2.76[2.48–3.06]	0.71[0.62–0.81]	0.62[0.50–0.76];	< 0.0001
All-cause mortality						
DPP4I users	128,619.7	1257	9.77[9.24–10.3]	1 [Reference]	1 [Reference]	NA
SGLT2I users	129,947.5	703	5.40[5.02–5.83]	0.55[0.50–0.61]	0.77[0.66–0.89];	0.0003

Adjusted for significant demographics, past comorbidities, non-SGLT2I/DPP4I medications, abbreviated MDRD, NLR, fasting glucose, HbA1c, and duration from earliest diabetes mellitus date to initial drug exposure date

*HR* hazard ratio, *CI* confidence interval, *SGLT2I* sodium glucose cotransporter-2 inhibitor, *DPP4I* dipeptidyl peptidase-4 inhibitor

### Association between SGLT2I and DPP4I and gastric diseases

For peptic ulcer, 103 SGLT2I users and 211 DPP4I users developed peptic ulcer. After a total follow-up of 257,427 person-year, the incidence of peptic ulcer was lower amongst SGLT2I users (IR per 1000 person-year: 0.80; 95% CI 0.65–0.96) compared to DPP4I users (IR per 1000 person-year: 1.65; 95% CI 1.44–1.89) with a rate ratio of 0.48 (95% CI 0.38–0.61) (Table 2). SGLT2I use was associated with a lower risk of peptic ulcer after adjustment (HR: 0.66; 95% CI 0.47–0.91, p = 0.0118) compared to DPP4I use (Fig. 2; Supplementary Fig. 3; Supplementary Table 3).

61 SGLT2I users and 185 DPP4I users developed acute gastritis during the follow-up period. After a total follow-up of 257,811.1 person-year, the incidence of acute gastritis was lower amongst SGLT2I users (IR per 1000 person-year: 0.47; 95% CI 0.36–0.60) compared to DPP4I users (IR per 1000 person-year: 1.44; 95% CI 1.24–1.67) with a rate ratio of 0.33 (95% CI 0.24–0.43) (Table 2). SGLT2I use was associated with a 69% lower risk of acute gastritis after adjustment (HR 0.31; 95% CI 0.20–0.48, p < 0.0001) compared to DPP4I use (Fig. 2; Supplementary Fig. 3; Supplementary Table 3).

For non-acute gastritis, 79 SGLT2I users and 246 DPP4I users developed non-acute gastritis. After a total follow-up of 257,547.2 person-year, the incidence of non-acute gastritis was lower amongst SGLT2I users (IR per 1000 person-year: 0.61; 95% CI 0.48–0.76) compared to DPP4I users (IR per 1000 person-year: 1.92; 95% CI 1.69–2.18) with a rate ratio of 0.31 (95% CI 0.25–0.41) (Table 2). SGLT2I use was associated with a 65% lower risk of non-acute gastritis after adjustment (HR 0.35; 95% CI 0.25–0.49, p < 0.0001) compared to DPP4I use (Fig. 2; Supplementary Fig. 3; Supplementary Table 3).

For GERD, 356 SGLT2I users and 395 DPP4I users developed GERD. After a total follow-up of 255,922.1 person-year, the incidence of GERD was lower amongst SGLT2I users (IR per 1000 person-year: 2.76; 95% CI 2.48–3.06) compared to DPP4I users (IR per 1000 person-year: 3.90; 95% CI 3.56–4.25) with a rate ratio of 0.71 (95% CI 0.62–0.81) (Table 2). SGLT2I use was associated with a lower risk of GERD after adjustment (HR 0.62; 95% CI 0.50–0.76, p < 0.0001) compared to DPP4I use (Fig. 2; Supplementary Fig. 3; Supplementary Table 3).

### Mortality outcomes

**Fig. 3:**
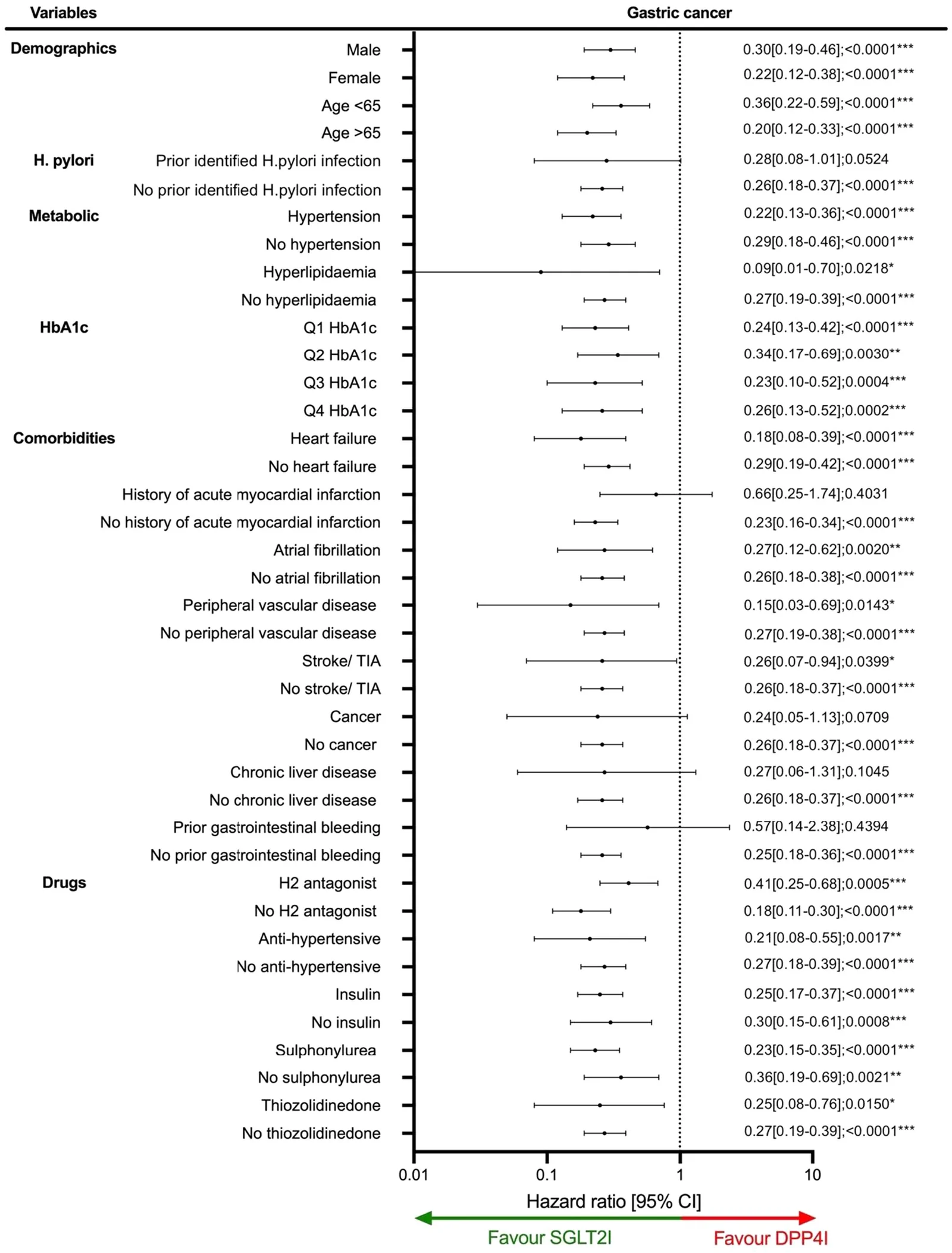

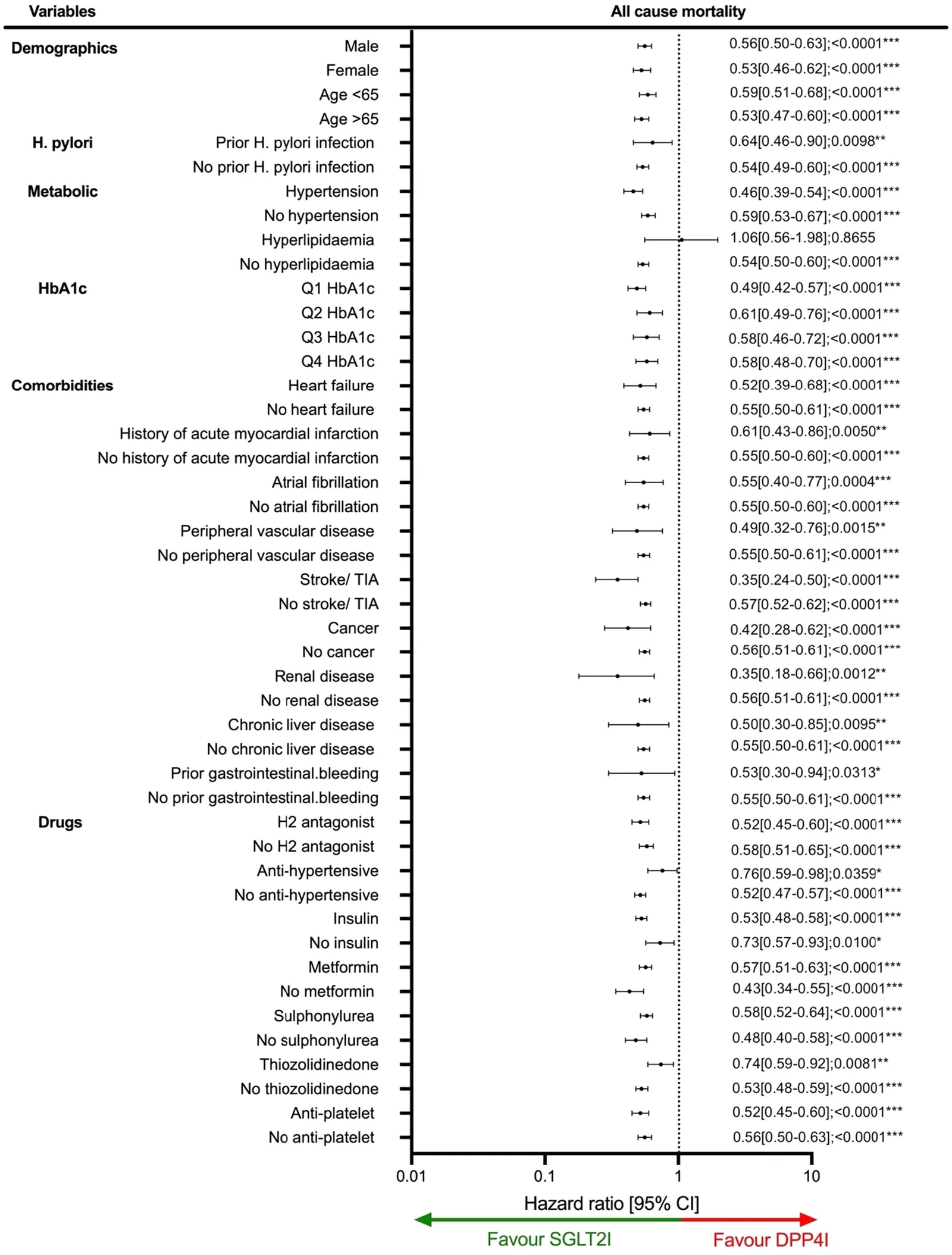

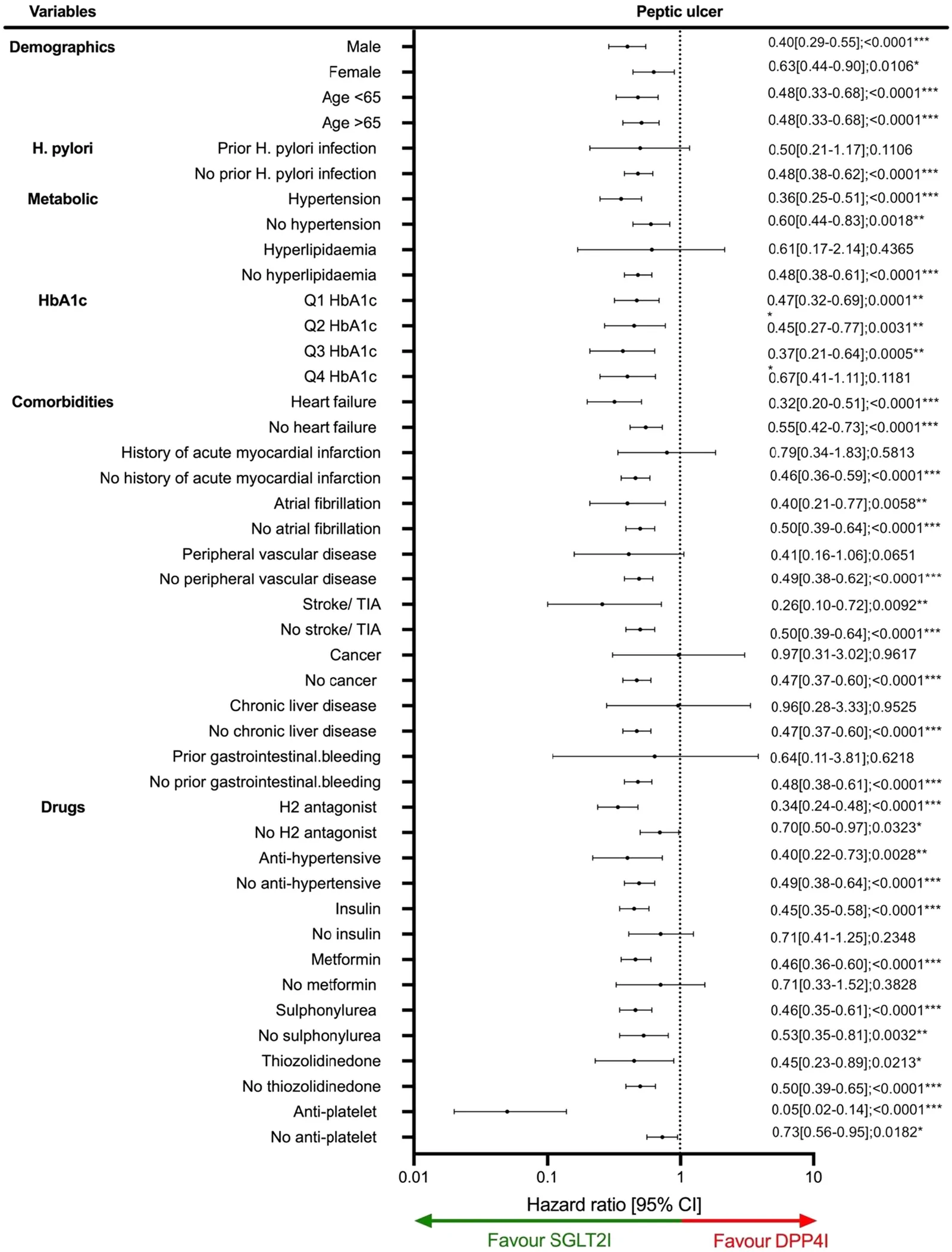

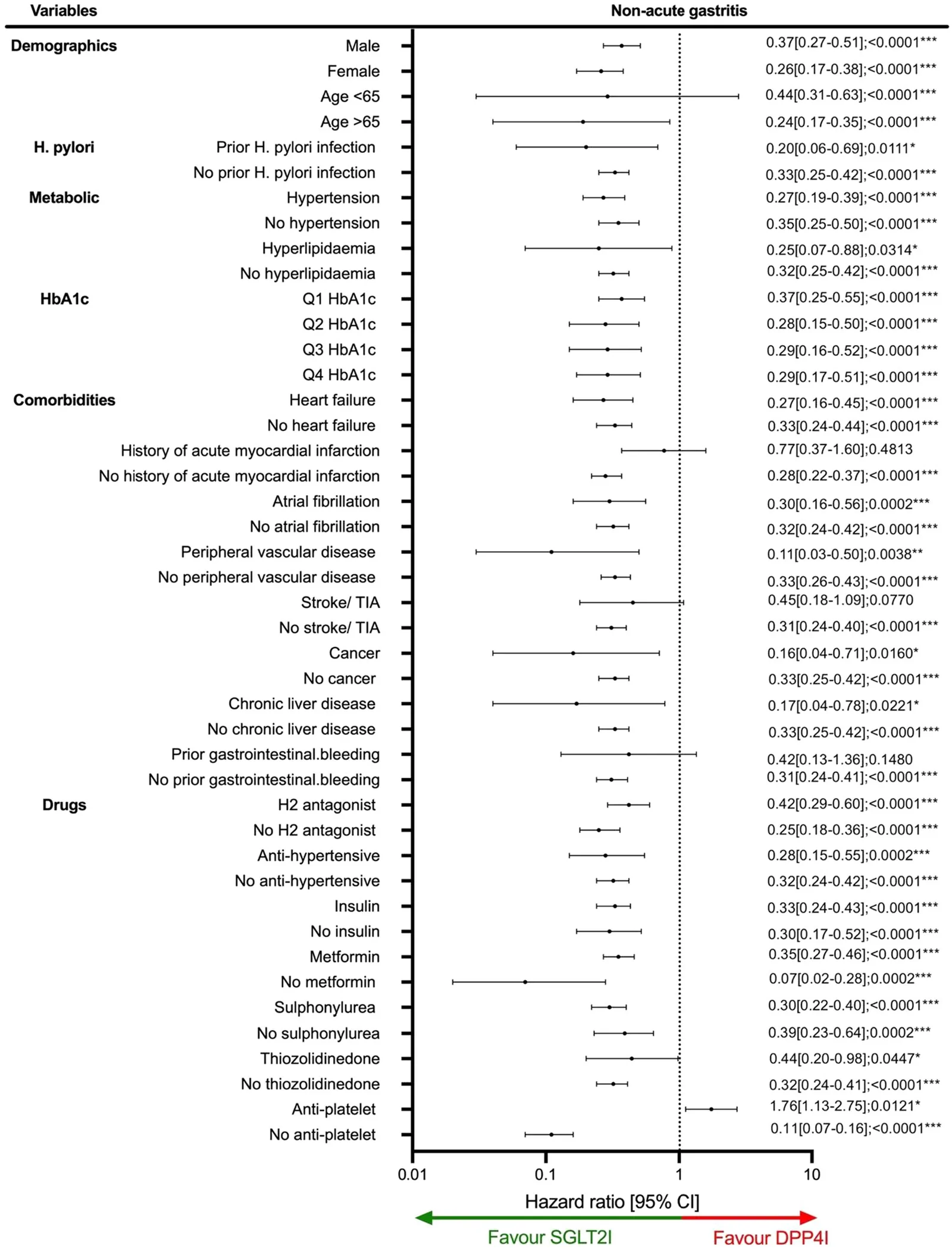

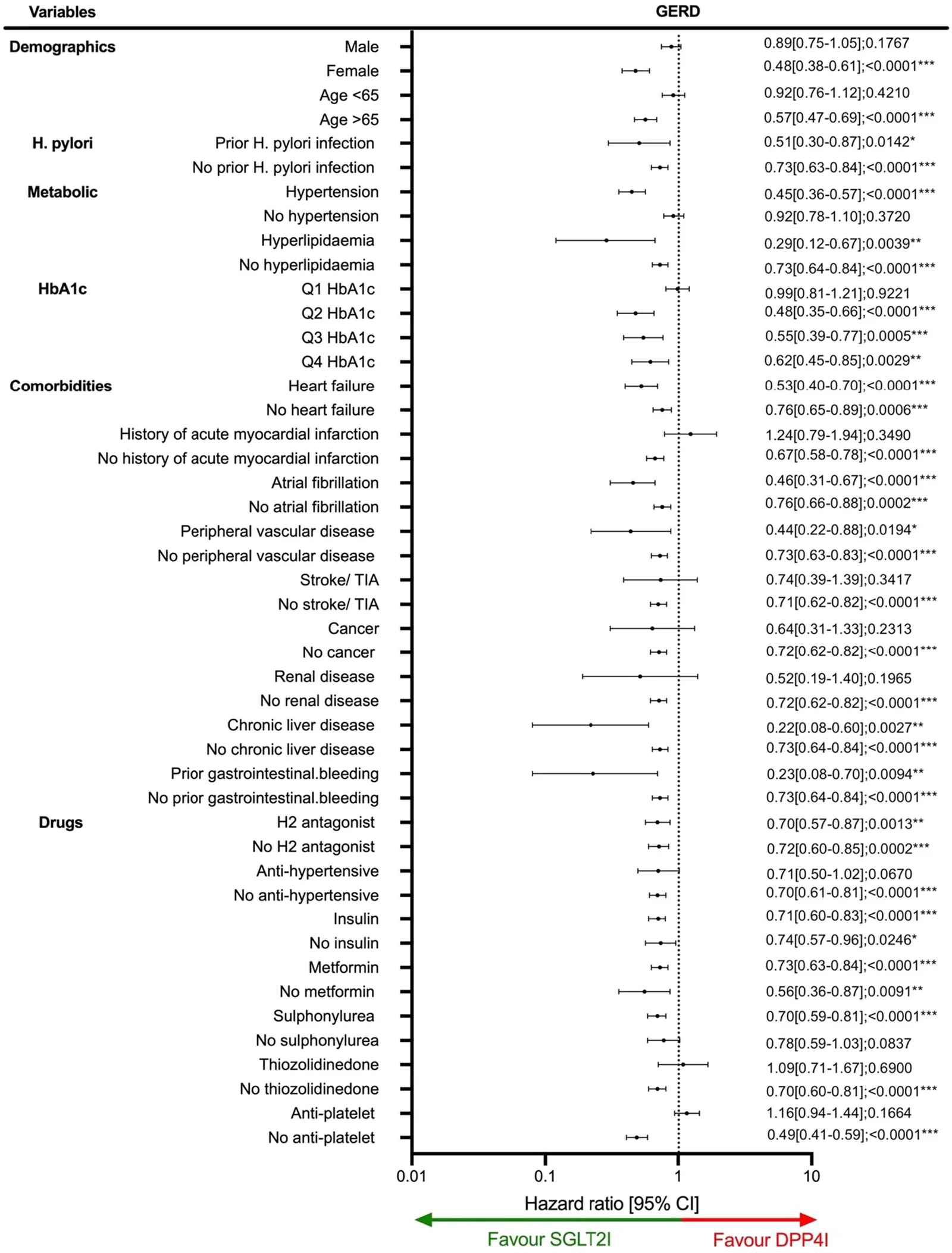

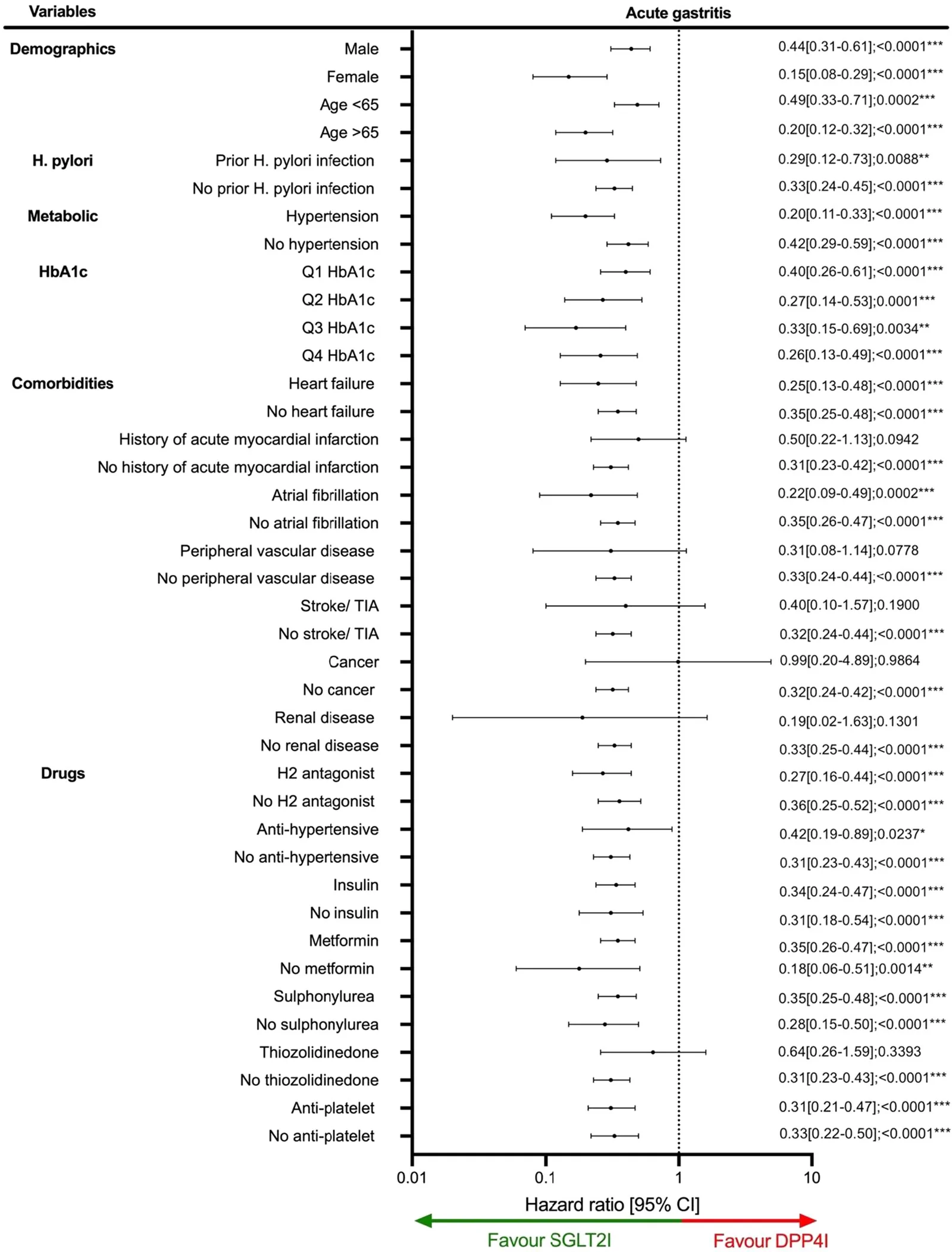
Subgroup analyses for SGLT2I v.s. DPP4I exposure predict new onset gastric cancer, gastric diseases, and all-cause mortality in the matched cohort. *SGLT2I* Sodium-glucose cotransporter-2 inhibitors, *DPP4I* Dipeptidyl peptidase-4 inhibitors, *Q1* Quartile 1, *Q2* Quartile 2, *Q3* Quartile 3, *Q4* Quartile 4, *TIA* Transient ischaemic attack, *CI* Confidence interval

703 SGLT2I users and 1257 DPP4I users passed away. After a follow-up of 258,567.2 person-year, the incidence of all-cause mortality was lower amongst SGLT2I users (IR 5.40; 95% CI 5.02–5.83) compared to DPP4I users (IR 9.77; 95% CI 9.24–10.3) with a rate ratio of 0.26 (95% CI 0.18–0.37) (Table 2). SGLT2I use was associated with a 70% lower risk of all-cause mortality after adjustment (HR 0.55; 95% CI 0.50–0.61, p < 0.0001) compared to DPP4I use regardless of the duration of diabetes mellitus (Supplementary Table 3). This was substantiated by the cumulative incidence curves stratified by SGLT2I versus DPP4I (Fig. 2; Supplementary Fig. 3). The marginal effect plotting HRs as a function of diabetes duration are shown in Supplementary Fig. 4, showing higher risks with longer disease duration. The results of the subgroup analysis for effects of SGLT2I and DPP4I uses on the gastric cancer and the gastric diseases are presented in Fig. 3 and Supplementary Figs. 5 to 6.

### Three-arm comparison between SGLT2I, DPP4I and GLP1a

A 3 arm analysis with the inclusion of GLP1a (included patients on SGLT2I, DPP4I, and GLP1a only) was conducted using stabilized IPTW (Supplementary Table 4 and 10). The bar charts of drug initialization dates for SGLT2I, DPP4I, and GLP1a uses are shown in Supplementary Fig. 7. The results between DPP4I and SGLT2I remained consistent with the main result (all p < 0.05) (Table 2). GLP1a use was associated with a 147% higher risk of gastric cancer (HR 2.47; 95% CI 1.27–4.81, p = 0.0079) after adjustment compared to SGLT2I use. The result also demonstrated that GLP1a use was associated with a higher risk of GERD (HR 1.43; 95% CI 1.04–1.97, p = 0.0299), but not peptic ulcer (HR 1.57; 95% CI 0.80–3.05, p = 0.1864), acute gastritis (HR 1.03; 95% CI 0.49–2.19, p = 0.9343), and non-acute gastritis (HR 1.51; 95% CI 0.99–2.32, p = 0.5818) after adjustment compared to SGLT2I use. Furthermore, DPP4I use was associated with higher risks of all-cause mortality (HR 2.57; 95% CI 2.36–2.79, p < 0.0001), while GLP1a’s risks of all-cause mortality (HR: 0.84; 95% CI 0.61–1.15; p = 0.2659) was not significantly different from SGLT2I use.

### Sensitivity analysis

The results of the cause-specific hazard models, sub-distribution hazard models, and different propensity score approaches demonstrated that different models did not change the point estimates for both the primary and the secondary outcomes (all p < 0.05) (Supplementary Table 5). Furthermore, when patients with CKD stage 4/5 (eGFR < 30), peritoneal dialysis, or haemodialysis were excluded from the matched cohort, SGLT2I use remained associated with lower risks of all gastric outcomes compared to DPP4I use (Supplementary Table 6). The three-arm analysis for 1-year lag time also demonstrated the same trend (all p < 0.05) (Supplementary Table 7). Moreover, the as-treat approach was used to account for the effects of drug-switching or discontinuation, and it did not affect the results (all p < 0.05) (Supplementary Table 8).

### Falsification analysis

Venous thromboembolism was used as the negative control outcome in the falsification analysis for the comparison between SGLT2I and DPP4I (Supplementary Table 9). The result showed no significant difference in the risk of venous thromboembolism between SGLT2I and DPP4I uses after adjustments (HR 1.21; 95% CI 0.98–1.53, p = 0.1034).

## Discussion

In this territory-wide cohort study, we used real-world data to compare the relationship between SGLT2I versus DPP4I on gastric cancer and gastric diseases (peptic ulcer, acute gastritis, non-acute gastritis, and GERD). Our results demonstrated that SGLT2I use was associated with lower risk of gastric cancer, peptic ulcer, acute gastritis, non-acute gastritis, and GERD than DPP4I use. To the best of our knowledge, this was the first cohort study to investigate the association between the novel anti-diabetic drugs with gastric cancer and the gastric diseases.

### Comparison with previous studies – gastric cancer

Overall, the incidence rate of gastric cancer in this study closely aligned with the previous studies [29, 30]. A previous study found an incidence of 1.28 per 1000 population for gastric cancer in China [29]. Multiple population-based observational studies illustrated the protective effects of metformin on gastric cancer [31]. However, studies examining the association between the novel anti-diabetic medications and gastric cancer remain scarce. In our study, the IR of the gastric cancer was the lower for SGLT2I users (0.32[0.23–0.43]) compared to DPP4I users (1.22 [1.03–1.42]). We also found similar rates for GLP1a use in the three-arm analysis.

The possible associations between SGLT2I or DPP4I use and gastric cancer risk remain controversial as current literature demonstrates conflicting evidence. There have been some promising evidence revealing decreased risks of gastric cancer observed with canagliflozin use [8]. This is further supported by other meta-analyses that have highlighted the decreased risk of GI cancer upon SGLT2I usage [32, 33]. The inhibitory effects of canagliflozin on SGLT1 and SGLT2 receptors can prevent glucose uptake of cancer cells, thereby increasing cell necrosis and reducing tumour growth [9]. SGLT2Is are also thought to enhance insulin sensitivity and diminish chronic inflammation, which may help mitigate the inflammatory microenvironment in T2DM that facilitates cancer development [10–12]. Given the relatively short follow-up duration of our study compared to the duration of gastric carcinogenesis, we hypothesised that the effects from the SGLT2I may not be totally arresting the carcinogenesis, but rather, slowing down the processes of carcinogenesis by the inhibitory mechanisms aforementioned.

A separate meta-analysis demonstrated that there was no significant increase in digestive system cancer risk observed in DPP4I use compared to placebo (RR 0.93 [0.77–1.13]) [34]. This is further corroborated by another study that show DPP4I usage was not associated with increased gastric cancer risk when comparing DPP4I, GLP1a and metformin usage [35]. In our study, the three-arm analysis found that DPP4I and GLP1a shared similar risks of gastric cancer compared to SGLT2I (Supplementary Table 4). Wong et al*.* revealed that adding DPP4I for diabetic patients on metformin-sulfonylurea therapy correlated with the lowest risk of overall cancer compared to insulin and thiazolidinediones [36]. This may be attributed to a combination of the immunological function of DPP4 in activating quiescent T-lymphocytes, leading to cell apoptosis and decreasing carcinogenesis [37]. However, it must be noted that there are currently no studies investigated the direct relationship between DPP4I and gastric cancer in T2DM patients. Several studies examined the protective effects of GLP1a against prostate, breast and cervical cancer [38–40]. The GLP1a cohort data was utilised as a form of additional analysis to confirm their overall effects on mortality given its relatively small sample size. As GLP1a is becoming more popular in Hong Kong, the GLP1a cohort will be expanded in few years’ time and its gastric effects can be further elucidated.

### Comparison with previous studies—gastric diseases

The incidence of peptic ulcers in this cohort is similar to the previous studies [41]. The current studies surrounding the influence of SGLT2I, DPP4I, and GLP1a on peptic ulcers is predominantly conducted through animal models. The SGLT2I dapagliflozin may decrease risk of peptic ulcer by lowering blood glucose and modulating ghrelin, motilin, and gastrin levels, thereby decreasing gastric acidity and inflammation while promoting mucosal healing [42]. On the other hand, DPP4I sitagliptin was effective against intestinal ulcers and improved ulcer healing through the activation of the GLP-2 signal pathway [43]. This effect cold be extrapolated to why anti-diabetic medications generate a protective effect against peptic ulcer.

A meta-analysis demonstrated conflicting results regarding the effect of SGLT2I, DPP4I and GLP1a on various types of gastritis. In general, no significant association between SGLT2I and DPP4I and gastric diseases was found, whereas some GLP1a medications were associated with increased risks of gastric diseases [44]. Furthermore, some studies elucidate that GLP1a usage was associated with increased risks of gastritis and GERD [45]. However, in an animal study, the use of empagliflozin was found to be associated with lower gastritis scores and milder inflammation compared to the control group [46].

Recent studies have found that SGLT2I and DPP4I agents can effectively improve GERD. Diabetic patients are at a higher risk of developing GERD due to autonomic neuropathy and obesity [47]. By promoting weight loss, diabetic medications can help mitigate obesity-related factors such as increased intragastric pressure, reduced lower esophageal sphincter pressure, and esophageal dysmotility, thus reducing the risk of GERD [48, 49]. Compared to Western countries, GERD is relatively less common in Asia, which parallels the incidence rate presented in this study cohort [50]. An analysis using Japanese adverse drug event report database (JADER) highlighting DPP4Is usage showed a lower incidence of GERD-like symptoms compared to GLP1a usage [51]. Meanwhile, a randomized controlled trial demonstrated that the use of lixisenatide and liraglutide did not have a significant impact on GERD or gastric motility [52].

### Clinical implications

The secondary protective benefits of the usage of SGLT2I and DPP4I on cardiovascular and gastric diseases have received worldwide attention in recent years. While more evidence supports the favourable effects of antidiabetic medications on cardiovascular mortality [53], there is limited evidence surrounding the latter. This notwithstanding, T2DM patients are susceptible to higher risk and mortality of gastric cancer [54, 55]. Therefore, the continuous elucidation of the possible implications of antidiabetic medications is crucial for optimising the management of gastric diseases, reducing healthcare resources and improving prognosis. In this study, we investigated the association using data from routine clinical practice, the result of which may influence the choice of second-line antidiabetic therapy in T2DM patients based on their gastric safety profile. As the results suggested, SGLT2I usage may be associated with a stronger protective effect against gastric cancer compared to DPP4I.

### Strengths and limitations

The strength of this study is the usage of CDARS. This comprehensive electronic health record database includes details of patient information such as drug prescription dates, time-serial laboratory results, and comorbidities. Hence, this limits information and selection and recall bias. The majority of current studies examined the effect of anti-diabetic medications as an individual predictor or compare two of the drugs. In contrast, our study compares two drugs with one extra drug (GLP1a) for a three-arm comparative analysis. Furthermore, only new users of SGLT2I and DPP4I were included in this study, so the effects of the baseline drug will be minimised. Lastly, to minimise the risk of residual confounding, we conducted a falsification analysis between SGLT2I and the DPP4I, which demonstrated that the use of SGLT2I was not associated with decreased risks of venous thromboembolism.

In contrast, several limitations should be acknowledged. First, due to the observational nature of this study, common variables, including smoking, drinking, BMI, and socioeconomic status, were also not available from CDARS. This could only be addressed by including the comorbidities and the laboratory test results in the study to indirectly refer to their cardiovascular risk factors. Propensity score matching and proportional hazard model were used to mitigate the effects of differences in baseline characteristics between SGLT2I and DPP4I users. Previous SGLT2I studies have also utilised a similar approach [25, 56], as well as non-SGLT2I pharmacoepidemiologic studies [57, 58]. However, since the cohorts were well-matched over a wide range of diseases and medications, and the falsification analysis suggested that SGLT2I was not associated with reduced risks of DPP4I, ideally, the covariates not included should be well-balanced. The data results may be susceptible to coding errors, missing data, under-coding and underreporting of clinical diagnoses. To minimise this, we have included all available data on laboratory tests and medications, and verified the ICD-9 diagnosis codes with existing studies. As the accurate medications and laboratory testing results were matched and also adjusted in the regression, the impacts of the coding error effects may be compensated.

Besides, the drug exposure duration, patient compliance to medication could not be standardised. The patient’s level of medication adherence was indirectly assessed through the frequency of prescription refills. This may lead to time lag biases and immortal time. Additionally, patients who may have switched between SGLT2I or DPP4I usage due to the presence of comorbidities or poor glycaemic control may have contributed to worse gastric outcomes among SGLT2I users. Nonetheless, this would result in an underestimation of the protective influences of SGLT2I against gastric outcomes and would not drastically impact the overall results.

Nevertheless, it was a common practice to get the ICD-9 code for patients with *H. pylori* infection diagnosed after oesophagogastroduodenoscopy (OGD). Hence, there may be an underestimation of *H. pylori* in ICD9 as not all patients may undergo OGD. Further research is warranted to explore the effects of serological gastritis markers and endoscopic findings. Furthermore, the nature of the study design suggests that the findings between specific drugs and gastric outcomes are correlational in nature. Thus, prospective randomised controlled trials are imperative to evaluate the causal links of anti-diabetic medications. Lastly, given the relatively low number of gastric cancers amongst the patients and the relatively short duration of follow-up compared to the gastric carcinogenesis process, despite a cohort of 257,947.7 person-year, the observation and the statistical outcome may require further follow-up in the future when SGLT2I are more frequently prescribed in the future.

## Conclusions

In this population-based cohort study, SGLT2I use was associated with lower risks of gastric cancer, peptic ulcer, acute gastritis, non-acute gastritis, and GERD compared to DPP4I use in the matched cohort on multivariable regression. These results may have potential clinical implications in reducing the gastric complications of T2DM. Further investigation into the mechanisms behind the association between SGLT2I use and gastric cancer is needed.

## Supplementary Information

Below is the link to the electronic supplementary material.Supplementary file1 (DOCX 9477 KB)

## Data Availability

Data are not available, as the data custodians (Hospital Authority) have not given permission for sharing due to patient confidentiality and privacy concerns. Local academic institutions, government departments, or nongovernmental organizations may apply for the access to data through the Hospital Authority’s data sharing portal (https://www3.ha.org.hk/data).

## Declarations

### Guarantor statement

All authors approved the final version of the manuscript. GT is the guarantor of this work and, as such, had full access to all the data in the study and takes responsibility for the integrity of the data and the accuracy of the data analysis.
