# Correction: Comparative effectiveness of sodium-glucose cotransporter-2 inhibitors for new-onset gastric cancer and gastric diseases in patients with type 2 diabetes mellitus: a population-based cohort study

**DOI:** 10.1007/s10120-026-01789-w

**Published:** 2026-08-31

**Authors:** Oscar Hou In Chou, Vinod Kumar Chauhan, Cheuk To Skylar Chung, Lei Lu, Teddy Tai Loy Lee, Zita Man Wai Ng, Karin Kai Wing Wang, Sharen Lee, Haipeng Liu, Ronald Ting Kai Pang, Apichat Kaewdech, Bernard Man Yung Cheung, Gary Tse, Jiandong Zhou

**Affiliations:** 1 Division of Clinical Pharmacology, Department of Medicine, School of Clinical Medicine, Li Ka Shing Faculty of Medicine, University of Hong Kong, Hong Kong, China; 2 Institute of Biomedical Engineering, Department of Engendering Science, University of Oxford, Oxford, UK; 3 Diabetes Research Unit, Cardiovascular Analytics Group, PowerHealth Research Institute, Hong Kong, China; 4 Research Centre for Intelligent Healthcare, Coventry University, Coventry, UK; 5 School of Nursing and Health Studies, Hong Kong Metropolitan University, Hong Kong, China; 6 Division of Internal Medicine, Faculty of Medicine, Prince of Songkla University, Hat Yai, Thailand; 7 Tianjin Key Laboratory of Ionic-Molecular Function of Cardiovascular Disease, Department of Cardiology, Tianjin Institute of Cardiology, Second Hospital of Tianjin Medical University, Tianjin, 300211 China; 8 Kent and Medway Medical School, Canterbury Christ Church University and University of Kent, Canterbury, UK; 9 Department of Family Medicine and Primary Care, Li Ka Shing Faculty of Medicine, The University of Hong Kong, Hong Kong, China; 10 School of Public Health, Li Ka Shing Faculty of Medicine, The University of Hong Kong, Hong Kong, China; 11 Department of Pharmacology and Pharmacy, The University of Hong Kong, Hong Kong, China

**Correction to: Gastric Cancer (2024) 27:947–970**

**https://doi.org/10.1007/s10120-024-01512-7**

In this article, the affiliation details were incorrect and revised in this correction. The original article has been corrected.

