# Supplementary material for: Comparative effectiveness of sodium-glucose cotransporter-2 inhibitors for new-onset gastric cancer and gastric diseases in patients with type 2 diabetes mellitus: a population-based cohort study
Source: Gastric Cancer. 2024 Jun 10;27(5):947–70. doi: 10.1007/s10120-024-01512-7 (PMC11335951; doi:10.1007/s10120-024-01512-7)
Supplement: Supplementary file 1 — Supplementary file1 (DOCX 9477 KB) [file 10120_2024_1512_MOESM1_ESM.docx]

**Supplementary Appendix** to

Comparative Effectiveness of Sodium-Glucose Cotransporter-2 Inhibitors for New-Onset Gastric Cancer and Gastric Diseases in Patients with Type 2 Diabetes Mellitus: A Population-Based Cohort Study

**Table of Contents**

[Supplementary Figure 1A: Distribution of date of T2DM diagnosis amongst the patients on SGLT2I, DPP4I, and GLP1a. 3](#_Toc159932617)

[SGLT2I: Sodium-glucose cotransporter-2 inhibitors; DPP4I: Dipeptidyl peptidase-4 inhibitors; Glucagon-like peptide-1 receptor agonist (GLP1a). 3](#_Toc159932618)

[Supplementary Figure 1B: Distribution of duration from medication initiation to outcome for SGLT2I, DPP4I, and GLP1a 4](#_Toc159932619)

[SGLT2I: Sodium-glucose cotransporter-2 inhibitors; DPP4I: Dipeptidyl peptidase-4 inhibitors; Glucagon-like peptide-1 receptor agonist (GLP1a). 4](#_Toc159932620)

[Supplementary Figure 1C: Bar chart of drug initialization dates for SGLT2I, DPP4I, and GLP1a in 2015. 5](#_Toc159932621)

[SGLT2I: Sodium-glucose cotransporter-2 inhibitors; DPP4I: Dipeptidyl peptidase-4 inhibitors; Glucagon-like peptide-1 receptor agonist (GLP1a). 5](#_Toc159932622)

[Supplementary Figure 2. Propensity score matching comparisons and proportional hazard assumption checking with parallel lines for SGLT2I v.s. DPP4I before and after 1:1 matching with nearest neighbor search strategy with calliper of 0.1 7](#_Toc159932623)

[Supplementary Figure 3. Cumulative incidence curves for new onset gastric outcomes and all-cause mortality stratified by drug exposure effects of SGLT2I and DPP4I before propensity score matching (1:1). 10](#_Toc159932624)

[Supplementary Figure 4. Marginal effects of diabetes duration with 95% Cis on new onset adverse gastric events and all-cause mortality stratified by drug use. 13](#_Toc159932625)

[Supplementary Figure 5A. Cumulative incidence curves for new onset gastric outcomes and all-cause mortality stratified by combinations of age and drug exposure effects of SGLT2I and DPP4I before propensity score matching. 16](#_Toc159932626)

[Supplementary Figure 5B. Cumulative incidence curves for new onset gastric outcomes and all-cause mortality stratified by combinations of age and drug exposure effects of SGLT2I and DPP4I in the matched cohort. 19](#_Toc159932627)

[Supplementary Figure 6A. Cumulative incidence curves for new onset gastric outcomes and all-cause mortality stratified by combinations of gender and drug exposure effects of SGLT2I and DPP4I before propensity score matching. 22](#_Toc159932628)

[Supplementary Figure 6B. Cumulative incidence curves for new onset gastric outcomes and all-cause mortality stratified by combinations of gender and drug exposure effects of SGLT2I and DPP4I in the matched cohort. 25](#_Toc159932629)

[Supplementary Table 1. The International Classification of Diseases, Clinical Modification (ICD-9-CM) codes for definitions of past comorbidities and outcomes. 26](#_Toc159932630)

[Supplementary Table 2. Calculations for variability measure 28](#_Toc159932631)

[Supplementary Table 3. Multivariable Cox regression models with adjustments to predict new gastric cancer and gastric diseases in the SGLT2I v.s. DPP4I matched cohort. 29](#_Toc159932632)

[Supplementary Table 4. Sensitivity analysis: Three-arm (only SGLT2I, only DPP4I, and only GLP1a) analysis results using stabilized IPTW. 30](#_Toc159932633)

[Supplementary Table 5. Sensitivity analyses: Exposure effects of SGLT2I v.s. DPP4I on new onset gastric diseases in the SGLT2I v.s. DPP4I matched cohort across different hazard models and matching methods. 31](#_Toc159932634)

[Supplementary Table 6. Sensitivity analysis: Excluding patients with CKD stage 4/5 (eGFR <30), peritoneal dialysis or haemodialysis in the SGLT2I v.s. DPP4I matched cohort. 32](#_Toc159932635)

[Supplementary Table 7. Sensitivity analysis: Consideration of 1-year lag time effects in the SGLT2I v.s. DPP4I matched cohort. 32](#_Toc159932636)

[Supplementary Table 8. Sensitivity analysis: As-treated approach of SGLT2I v.s. DPP4I on new onset gastric diseases in the SGLT2I v.s. DPP4I matched cohort 33](#_Toc159932637)

[Supplementary Table 9. Falsification analysis: Exposure effects of SGLT2I v.s. DPP4I on new onset venous thromboembolism in the SGLT2I v.s. DPP4I matched cohort after 1:1 propensity score matching 33](#_Toc159932638)

[Supplementary Table 10. The basic characteristics of the SGLT2I, DPP4I and GLP1a patients in the 3-arm analysis. 34](#_Toc159932639)

**
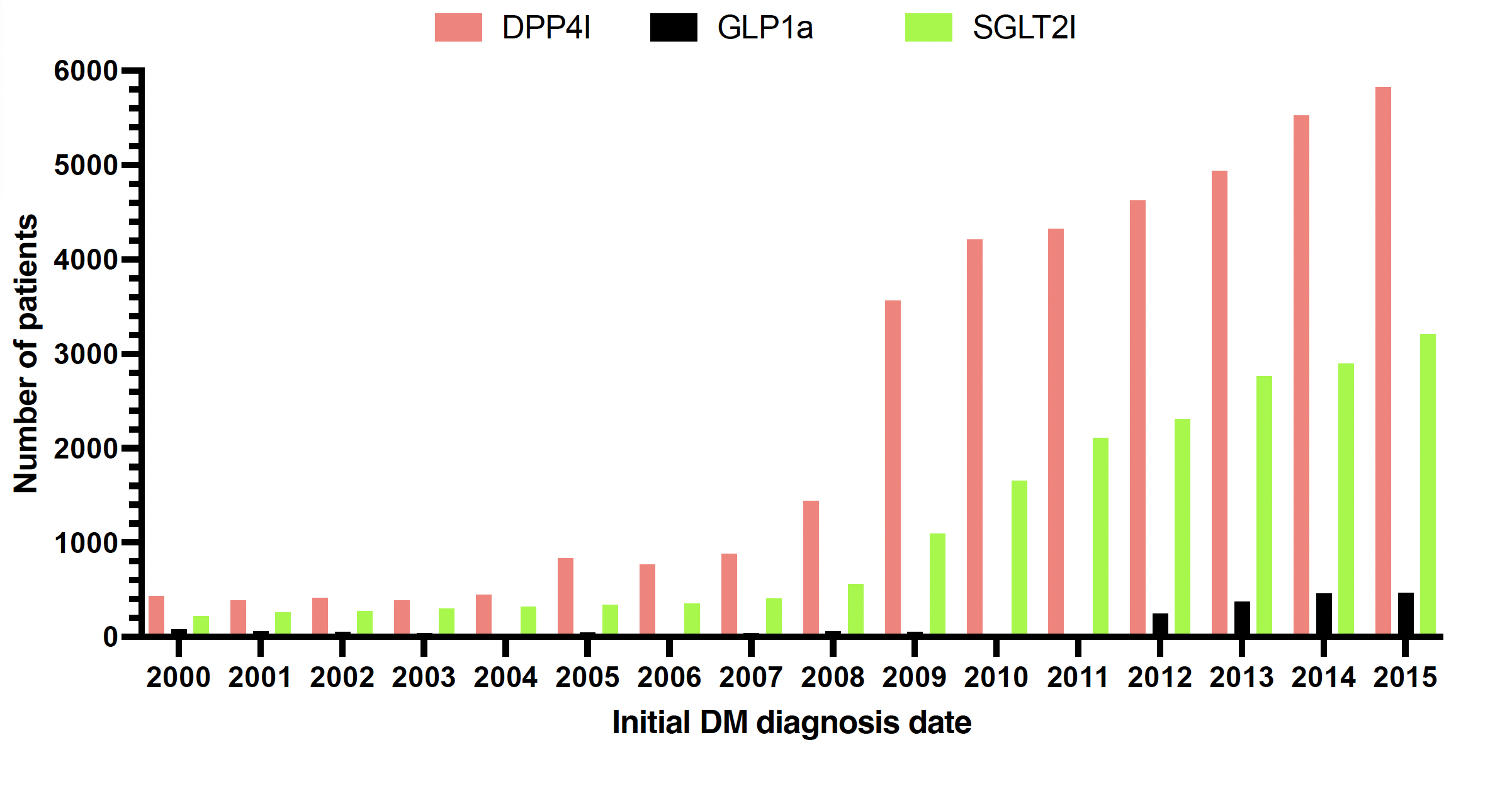
**

# Supplementary Figure 1A: Distribution of date of T2DM diagnosis amongst the patients on SGLT2I, DPP4I, and GLP1a.

# SGLT2I: Sodium-glucose cotransporter-2 inhibitors; DPP4I: Dipeptidyl peptidase-4 inhibitors; Glucagon-like peptide-1 receptor agonist (GLP1a).


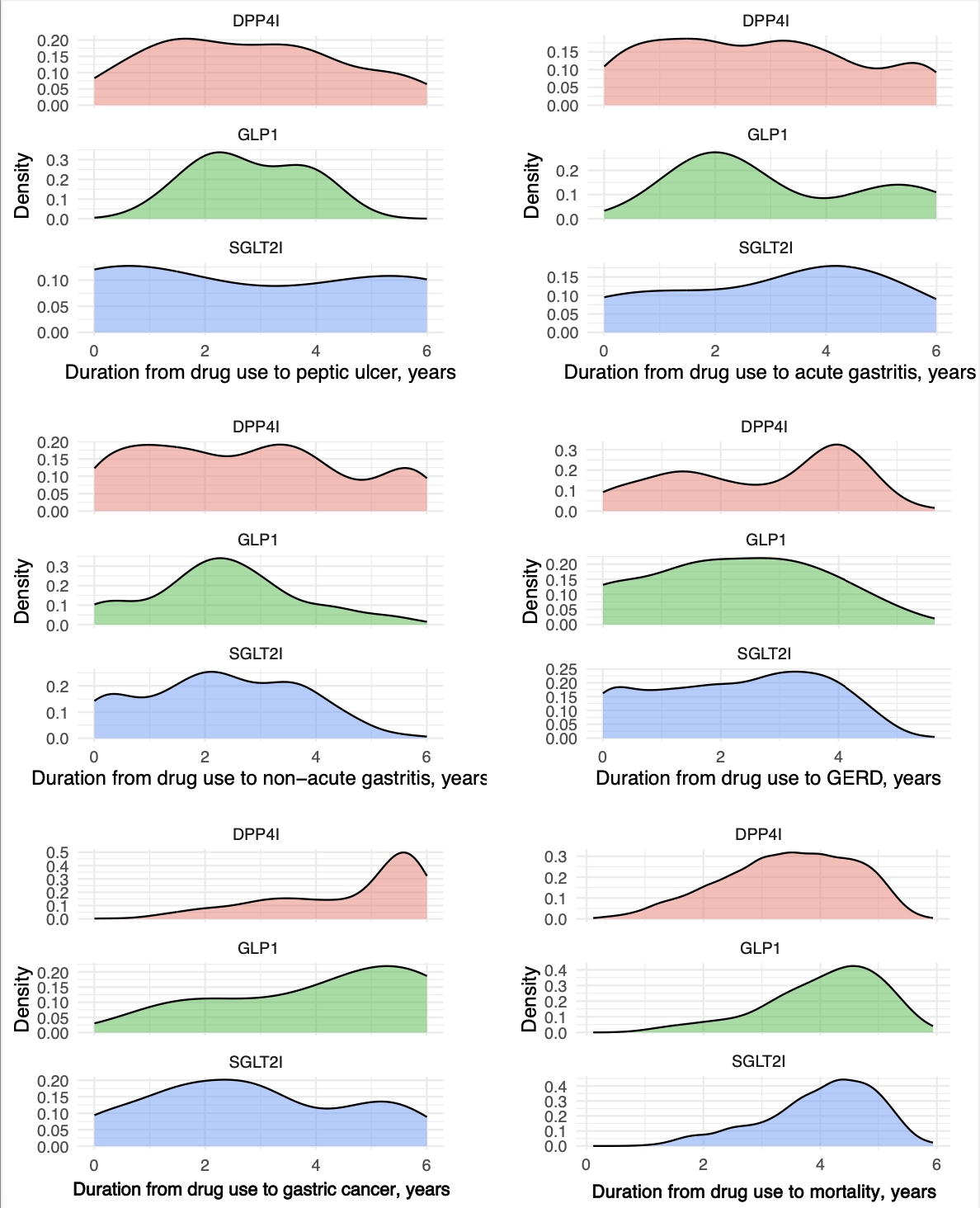


# Supplementary Figure 1B: Distribution of duration from medication initiation to outcome for SGLT2I, DPP4I, and GLP1a

# SGLT2I: Sodium-glucose cotransporter-2 inhibitors; DPP4I: Dipeptidyl peptidase-4 inhibitors; Glucagon-like peptide-1 receptor agonist (GLP1a).


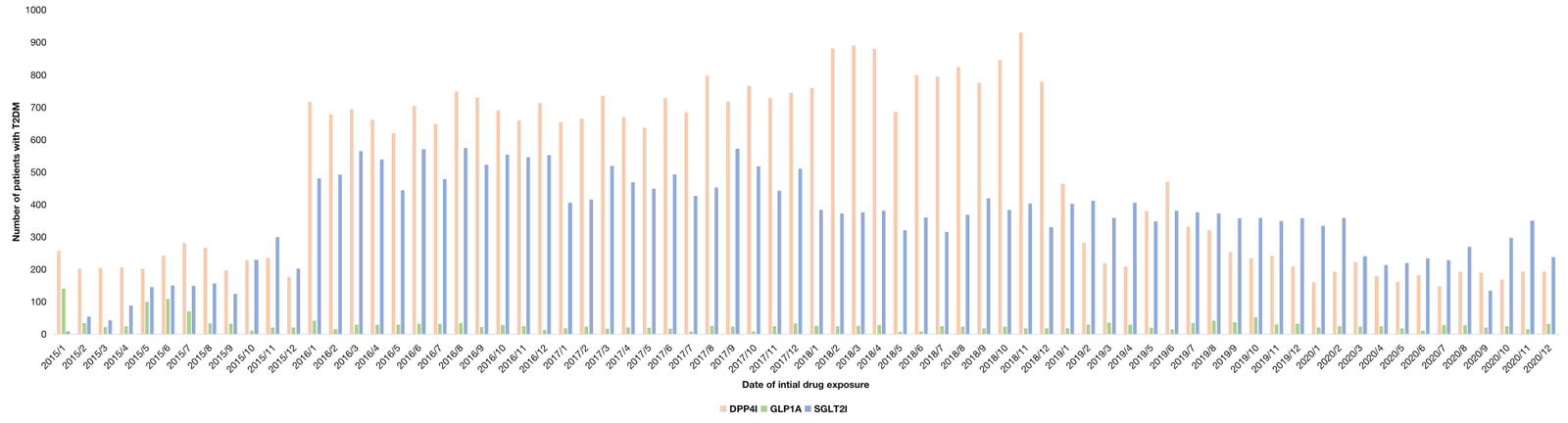


# Supplementary Figure 1C: Bar charts of the first prescription dates for the different drug classes of SGLT2I, DPP4I, and GLP1a.

# SGLT2I: Sodium-glucose cotransporter-2 inhibitors; DPP4I: Dipeptidyl peptidase-4 inhibitors; Glucagon-like peptide-1 receptor agonist (GLP1a).

# Supplementary Figure 2. Propensity score matching comparisons and proportional hazard assumption checking with parallel lines for SGLT2I v.s. DPP4I before and after 1:1 matching with nearest neighbor search strategy with calliper of 0.1

SGLT2I: Sodium-glucose cotransporter-2 inhibitors; DPP4I: Dipeptidyl peptidase-4 inhibitors.


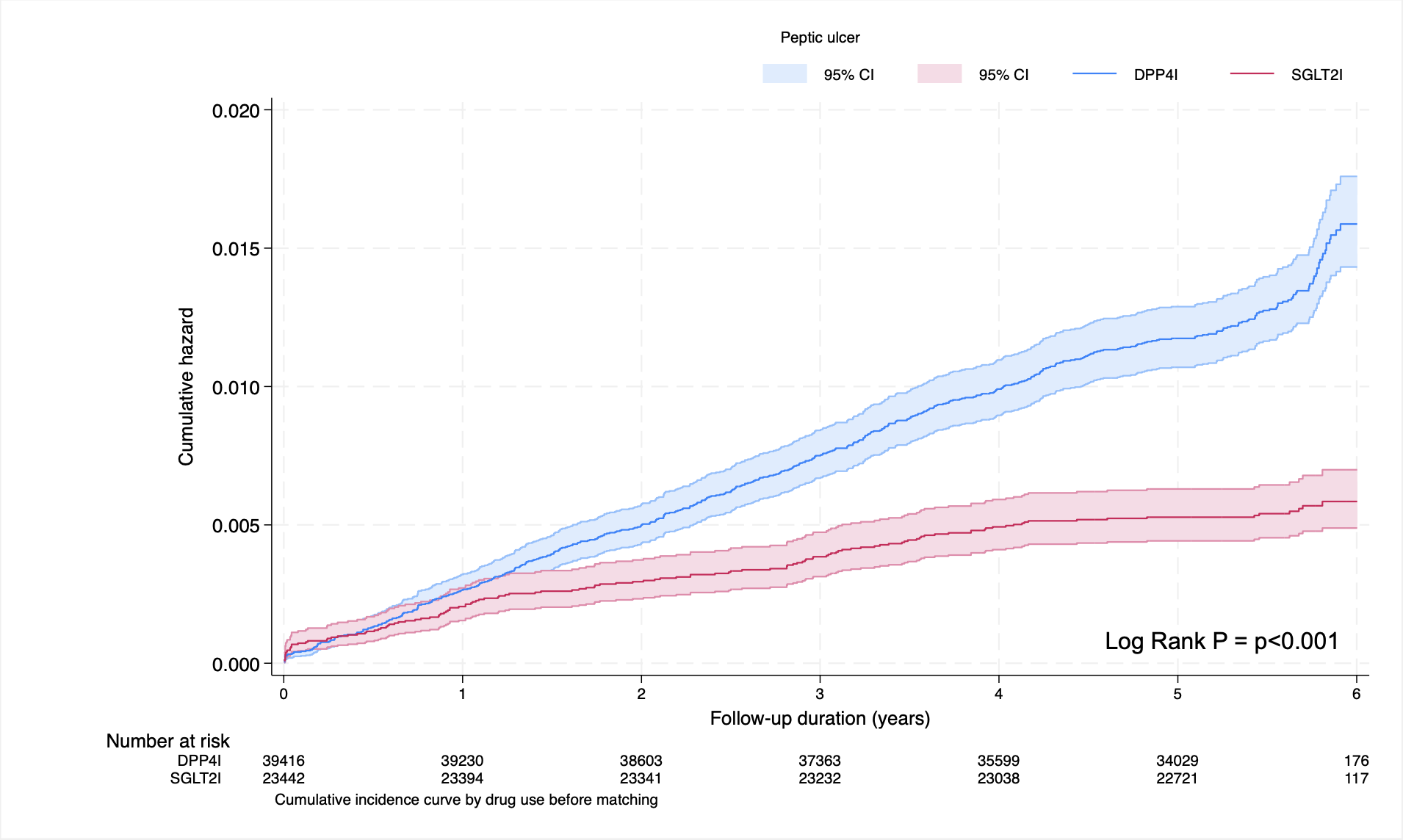

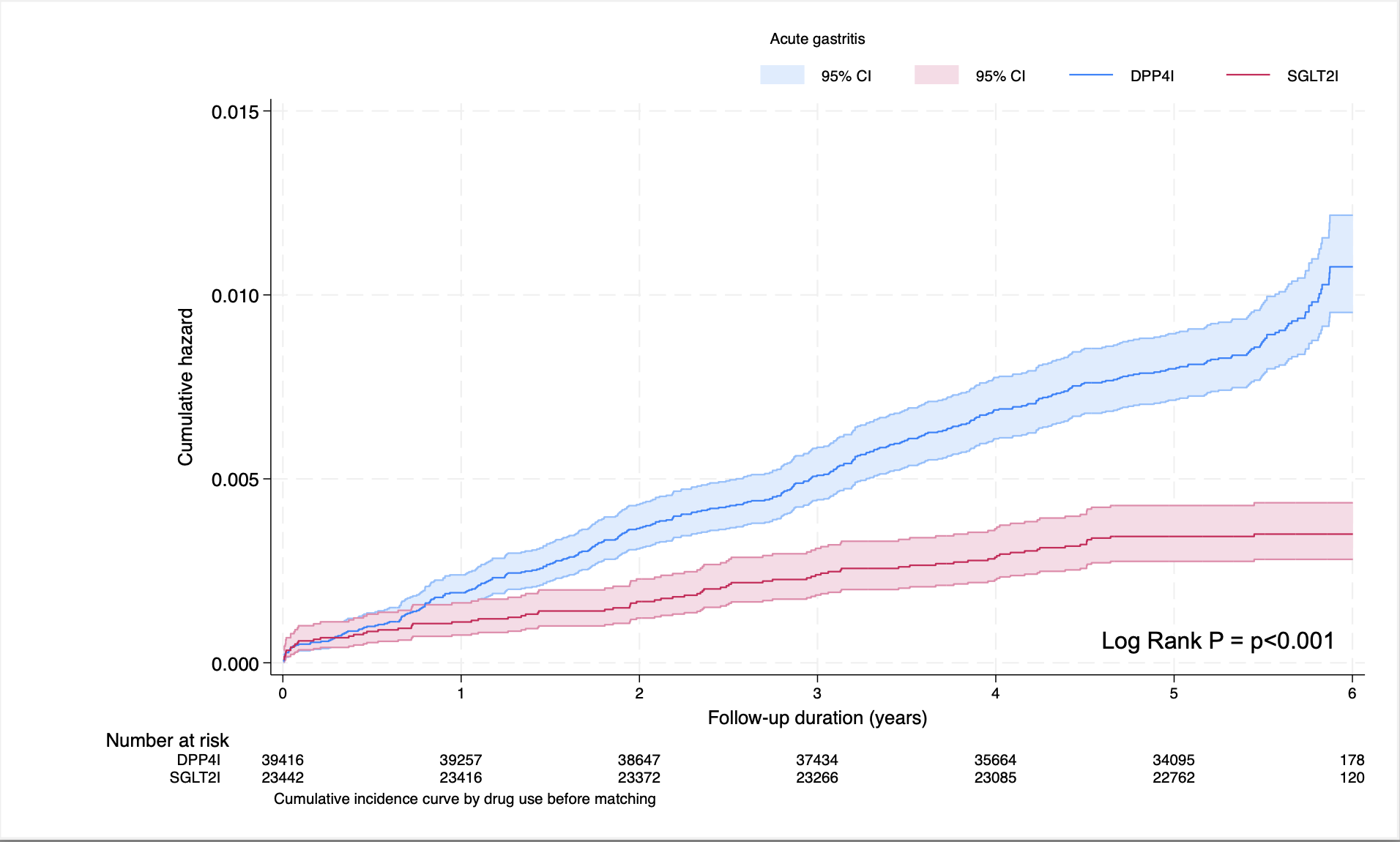

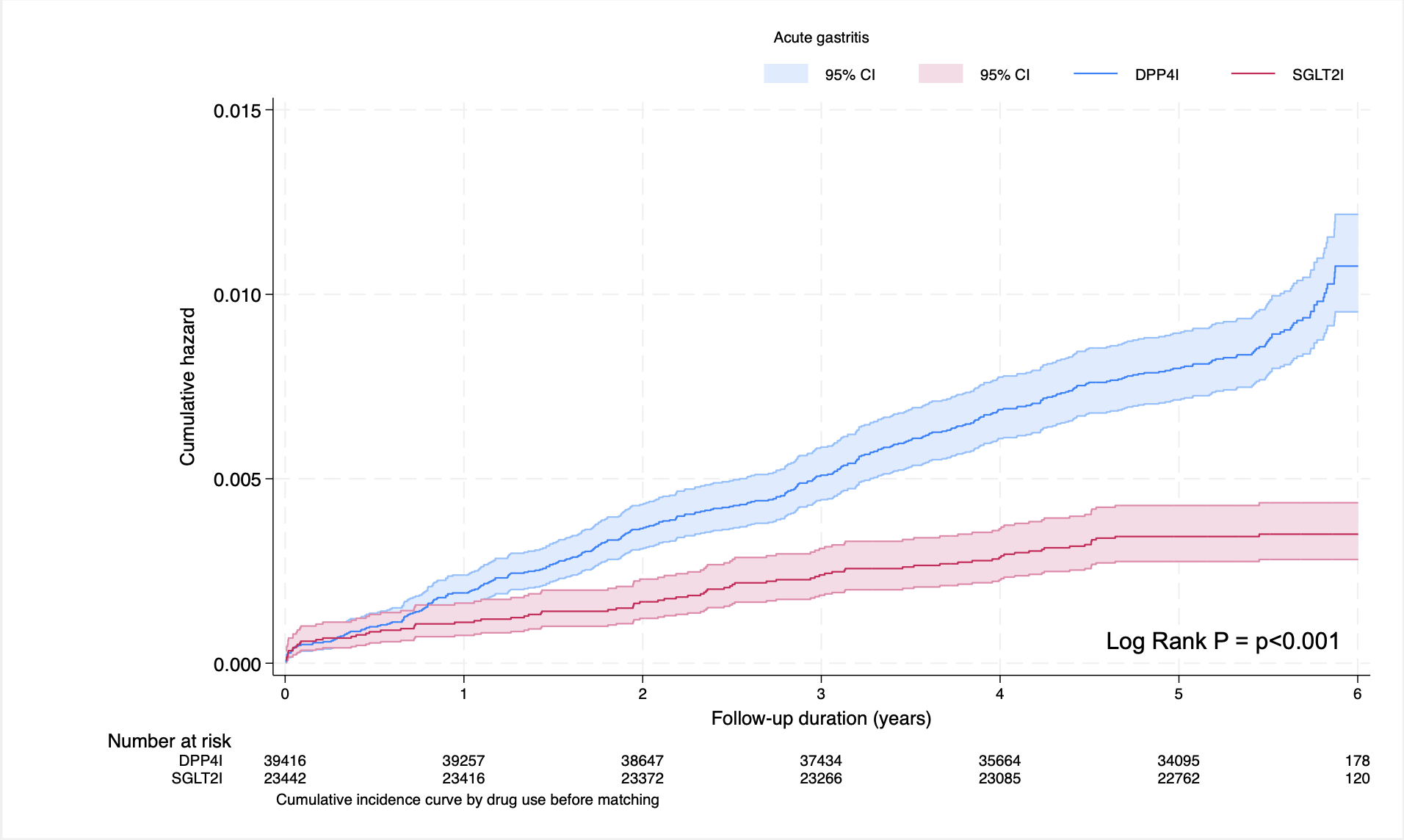

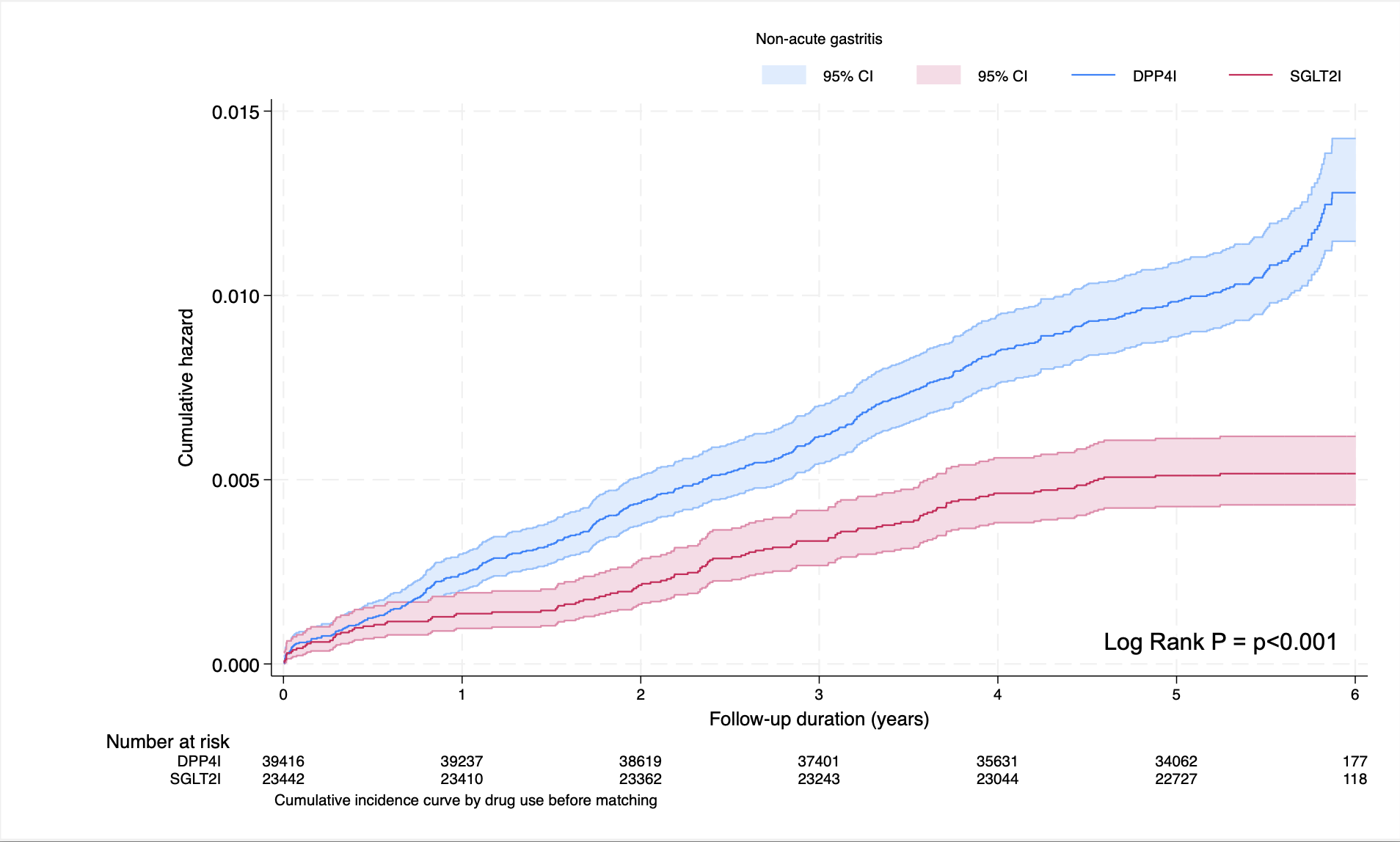

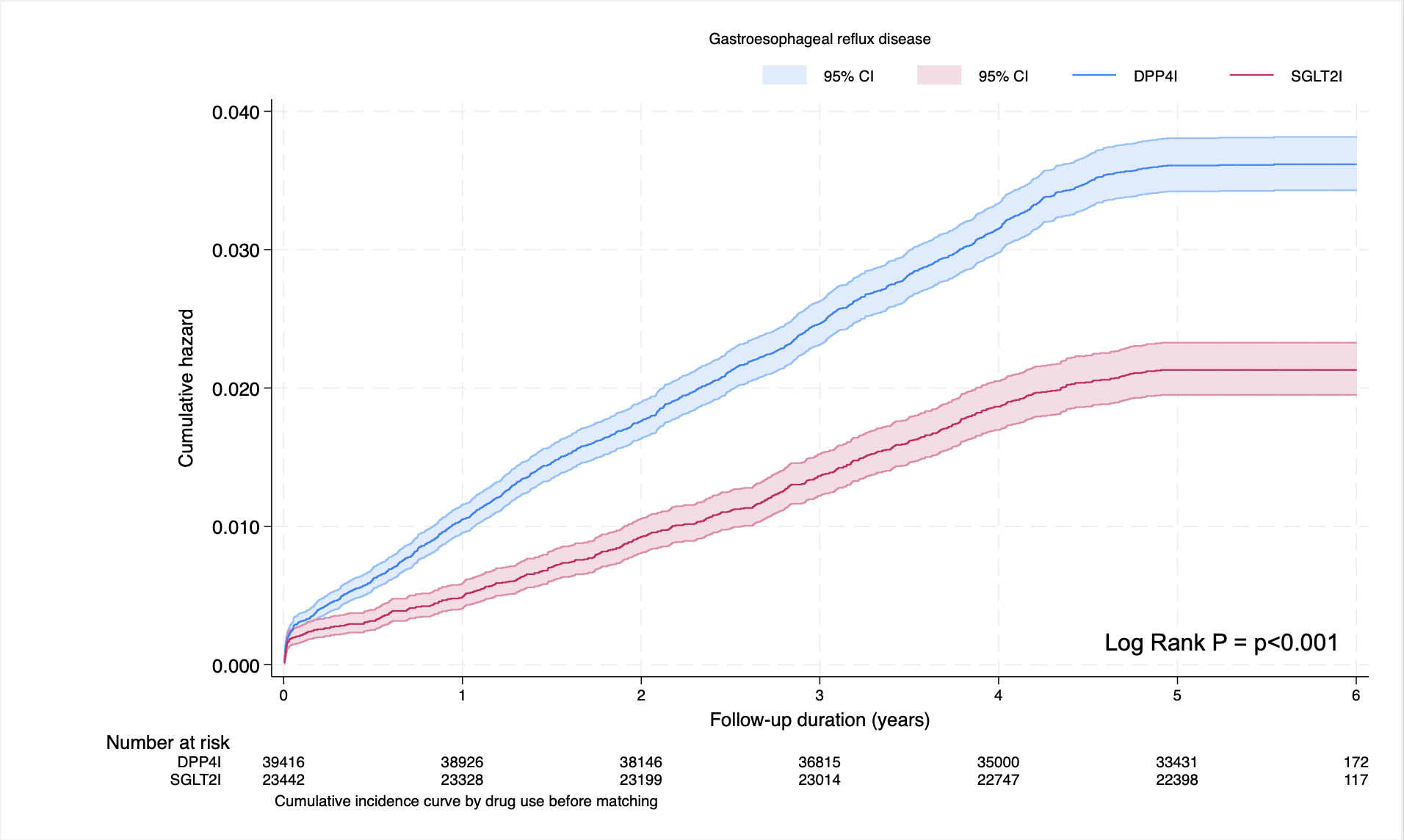

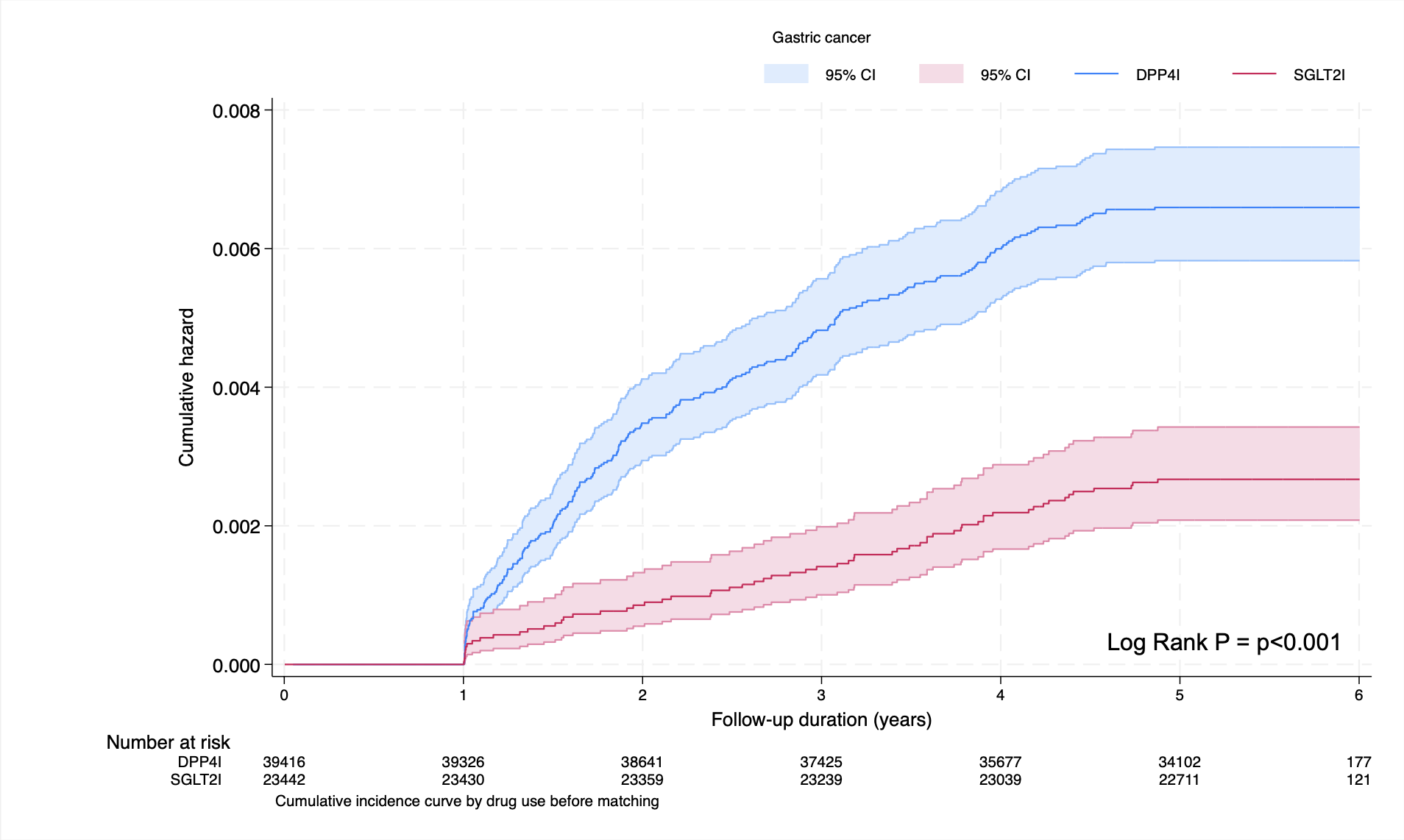

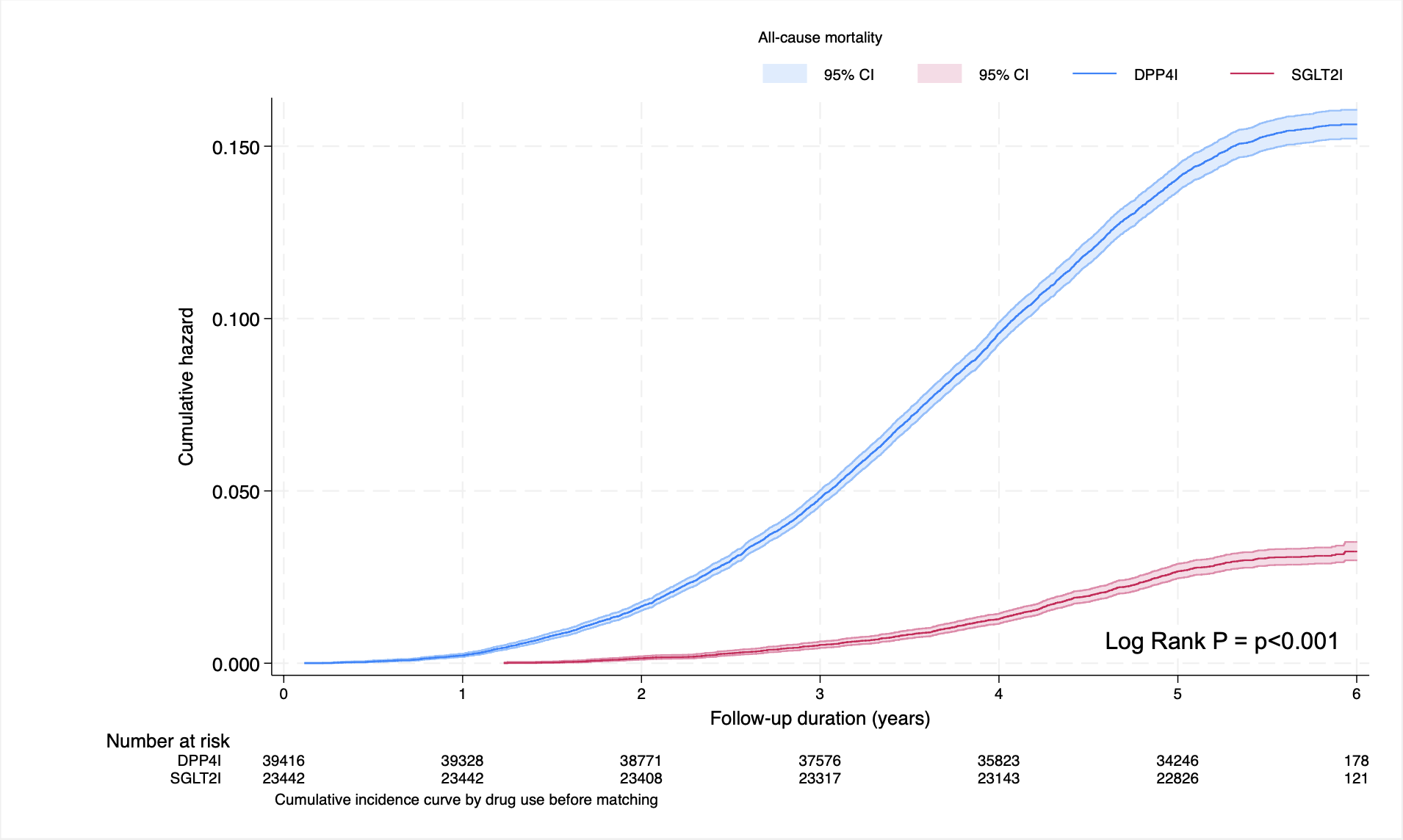


# Supplementary Figure 3. Cumulative incidence curves for new onset gastric outcomes and all-cause mortality stratified by drug exposure effects of SGLT2I and DPP4I before propensity score matching (1:1).

SGLT2I: Sodium-glucose cotransporter-2 inhibitors; DPP4I: Dipeptidyl peptidase-4 inhibitors.

# Supplementary Figure 4. Marginal effects of diabetes duration with 95% Cis on new onset adverse gastric events and all-cause mortality stratified by drug use.

SGLT2I: Sodium-glucose cotransporter-2 inhibitors; DPP4I: Dipeptidyl peptidase-4 inhibitors.


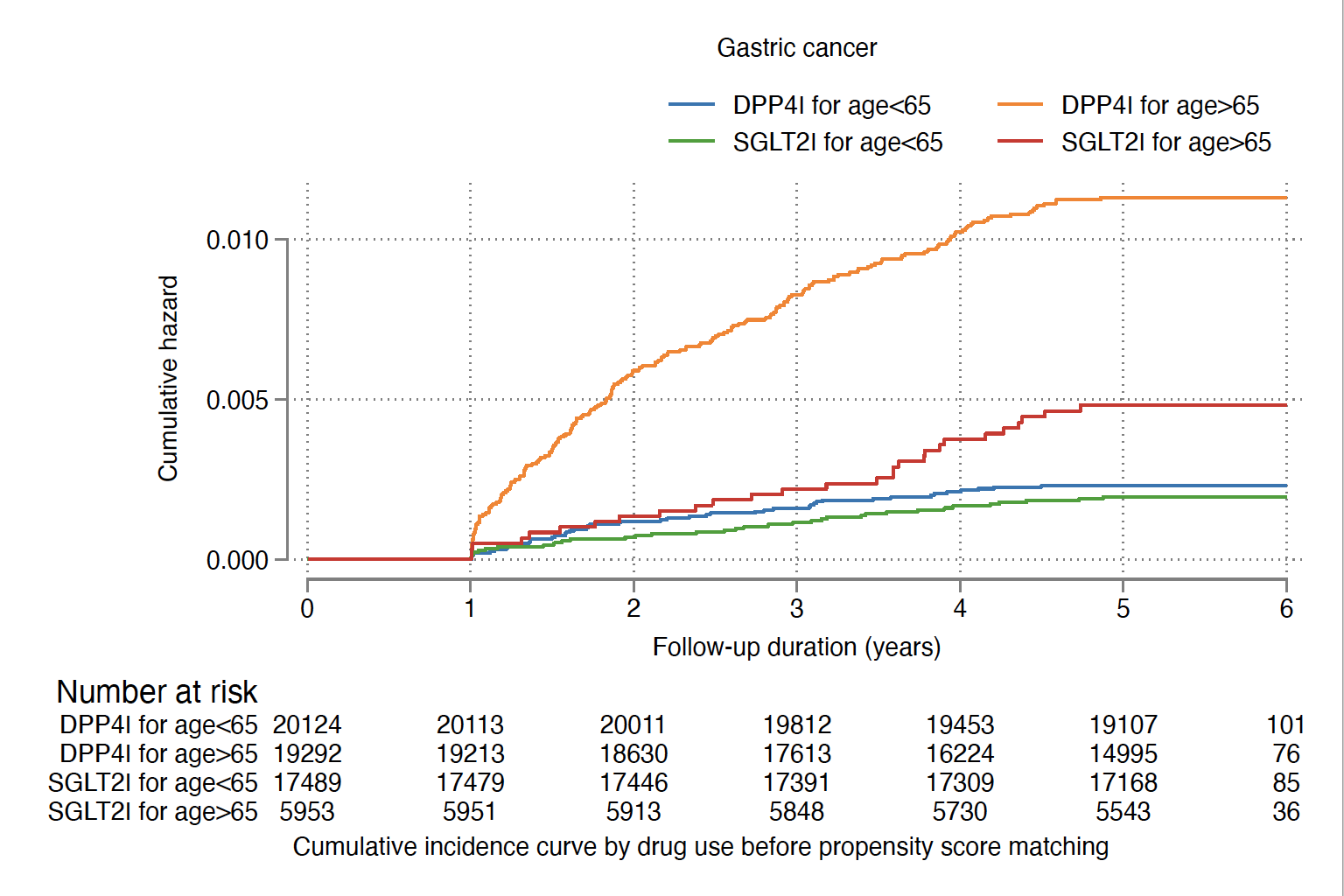


# Supplementary Figure 5A. Cumulative incidence curves for new onset gastric outcomes and all-cause mortality stratified by combinations of age and drug exposure effects of SGLT2I and DPP4I before propensity score matching.

SGLT2I: Sodium-glucose cotransporter-2 inhibitors; DPP4I: Dipeptidyl peptidase-4 inhibitors.

 **
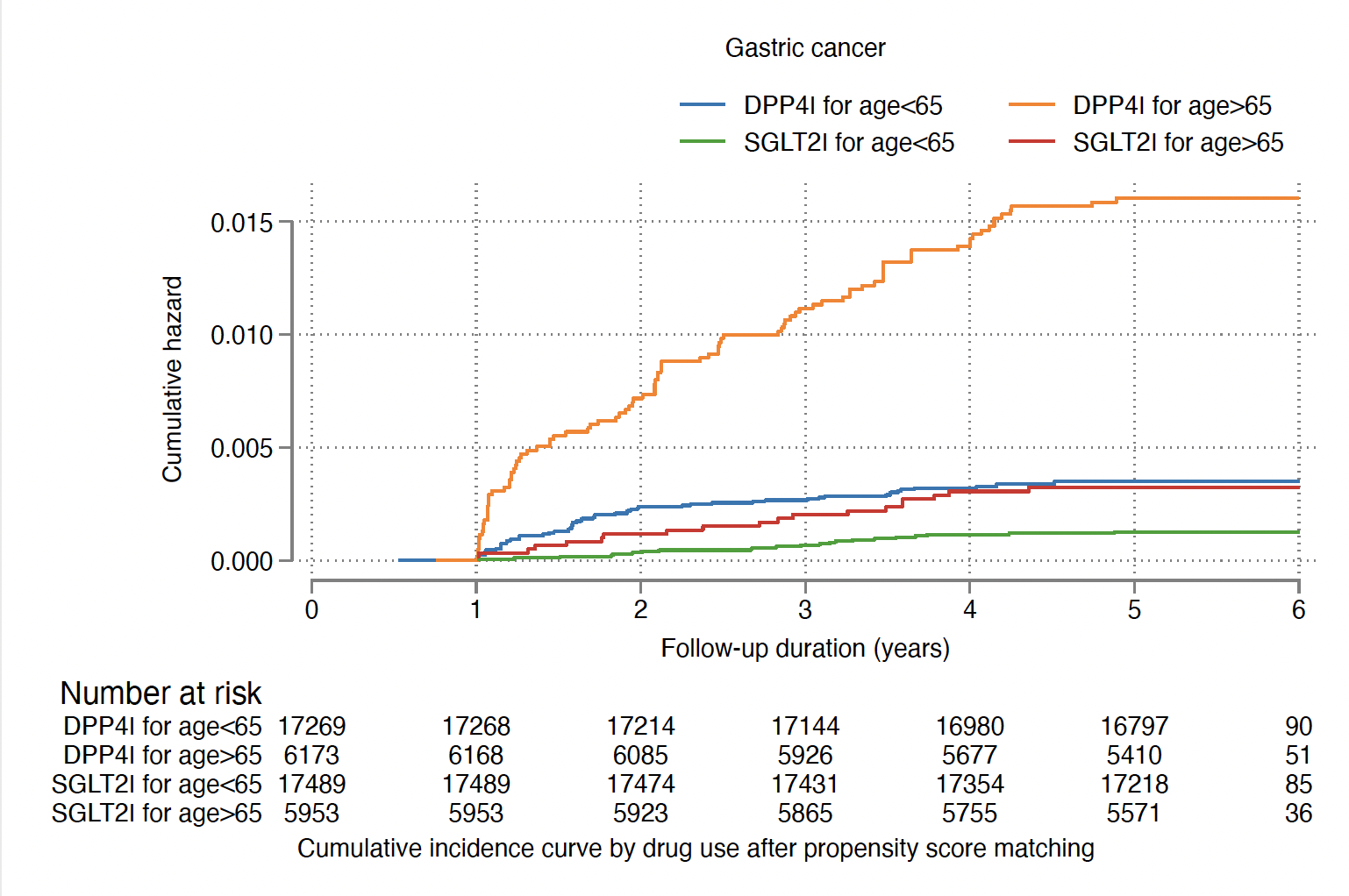
**

# Supplementary Figure 5B. Cumulative incidence curves for new onset gastric outcomes and all-cause mortality stratified by combinations of age and drug exposure effects of SGLT2I and DPP4I in the matched cohort.

SGLT2I: sodium glucose cotransporter-2 inhibitor; DPP4I: dipeptidyl peptidase-4 inhibitor

**** **
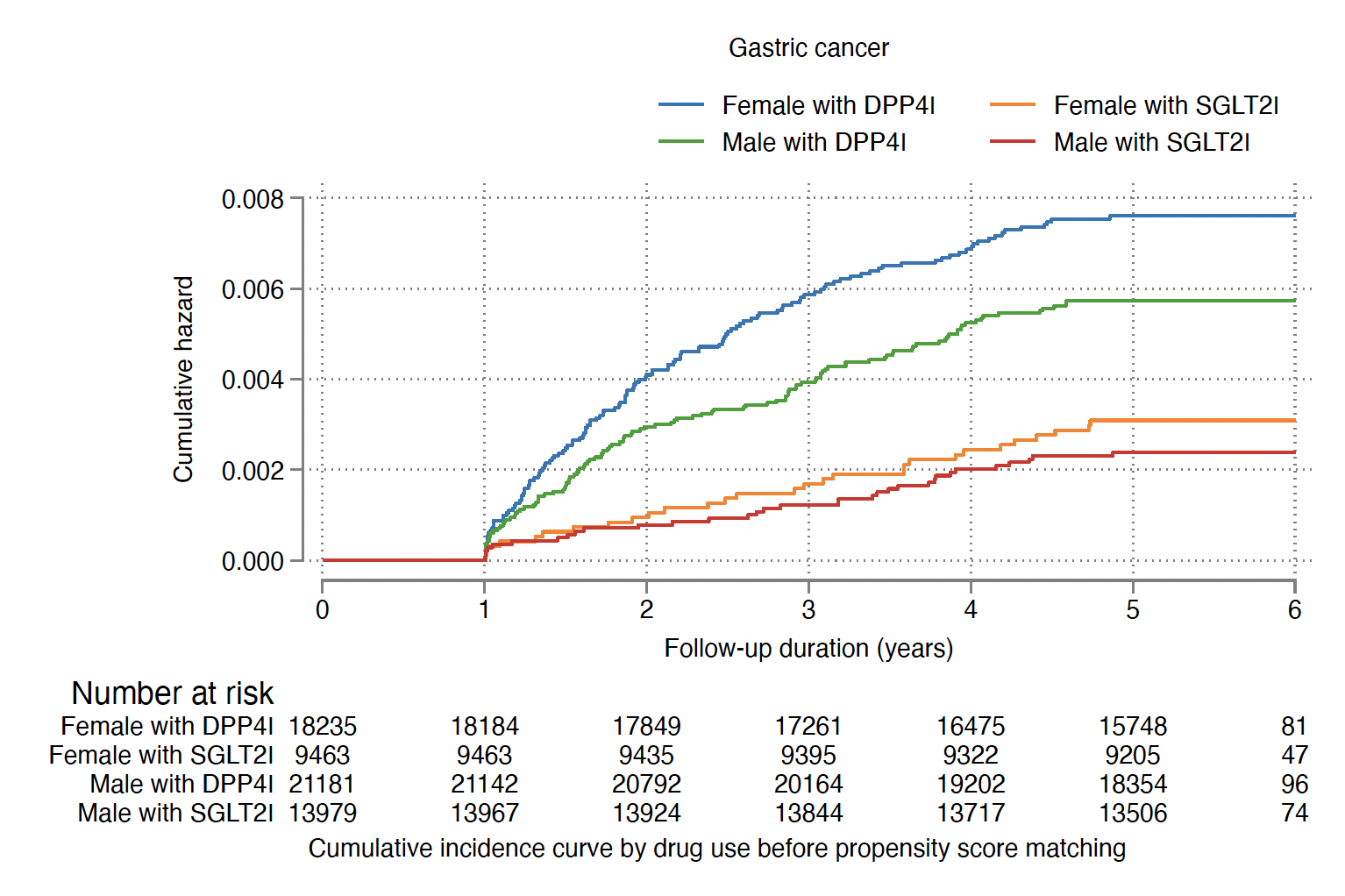
**

# Supplementary Figure 6A. Cumulative incidence curves for new onset gastric outcomes and all-cause mortality stratified by combinations of gender and drug exposure effects of SGLT2I and DPP4I before propensity score matching.

SGLT2I: Sodium-glucose cotransporter-2 inhibitors; DPP4I: Dipeptidyl peptidase-4 inhibitors.

**** **
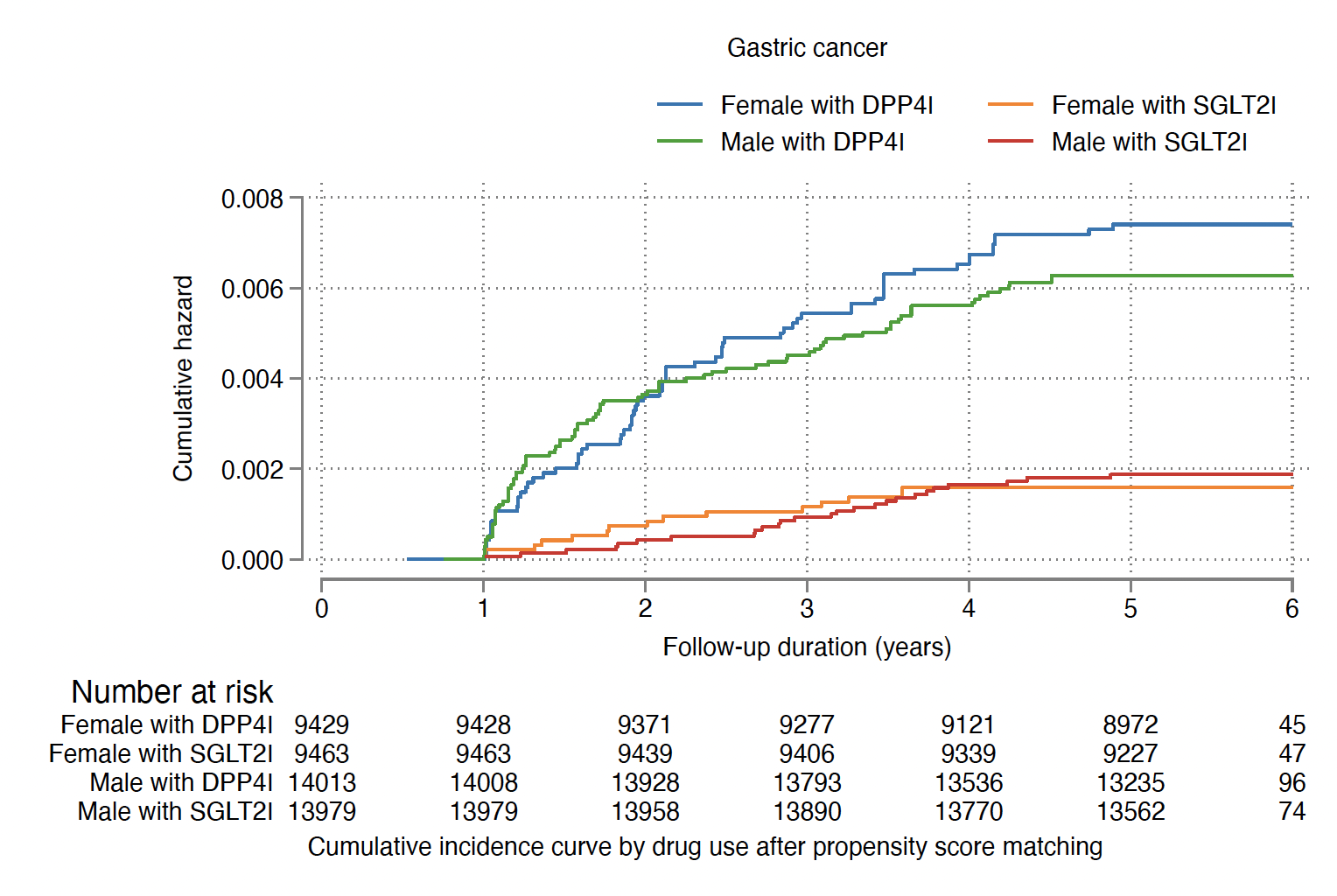
**

# Supplementary Figure 6B. Cumulative incidence curves for new onset gastric outcomes and all-cause mortality stratified by combinations of gender and drug exposure effects of SGLT2I and DPP4I in the matched cohort.

SGLT2I: Sodium-glucose cotransporter-2 inhibitors; DPP4I: Dipeptidyl peptidase-4 inhibitors.

# Supplementary Table 1. The International Classification of Diseases, Clinical Modification (ICD-9-CM) codes for definitions of past comorbidities and outcomes.

HBV: hepatitis B virus; HCV: hepatitis C virus

| **Adverse gastric outcomes of interest** |
| --- |
| **Peptic ulcer:** 531–533 |
| **Acute gastritis:** 535.0 |
| **Non-acute gastritis:** 535.1–535.4, 535.7 |
| **Gastroesophageal reflux disease:** 530.81, 530.11 |
| **Gastric cancer:** 151, 151.0, 151.1, 151.2, 151.3, 151.4, 151.5, 151.6, 151.8, 151.9, 230.2 |
| **Past comorbidities** |
| **Cancer**: 140-239, excluded gastric cancer |
| **Hypertension**: 401 401.1 401.9 402 402.01 402.1 402.11 402.9 402.91 403 403.01 403.1 403.11 403.9 403.91 404 404.01 404.02 404.03 404.1 404.11 404.12 404.13 404.9 404.91 404.92 404.93 405 405.01 405.09 405.1 405.11 405.19 405.9 405.91 405.99 437.2 |
| **Chronic liver disease and cirrhosis**: 571.0 571.1 571.2 571.3 571.40 571.41 571.42 471.49 571.5 571.6 571.8, 571.9 |
| **HBV infection:** 070.20–070.23, 070.30–070.33, V02.61 |
| **HCV infection:** 070.41, 070.44, 070.51, 070.54, 070.7, 070.70, 070.71 |
| **History of acute liver injury:** 570 572.2 |
| **Other liver disease:** 275.1 275.0 572.0 572.4 572.1 572.3 572.8 573.0 573.4 573.8 573.9 |
| **Autoimmune diseases:** 136.1, 359.79, 359.71, 443.1, 446, 555, 556.8~556.9, 556.0~556.6, 695.4, 710, 714, 720, 725 |
| **Polyps:** 569.0, 211.3 |
| **Gastrointestinal bleeding:** 578.9 |
| **Heart failure:** 428 428 428.1 428.2 428.2 428.21 428.22 428.23 428.3 428.3 428.31 428.32 428.33 428.4 428.4 428.41 428.42 428.43 428.9 398.91 402.01 402.11 402.91 404.01 404.03 404.11 404.13 404.91 404.93 |
| **Atrial fibrillation:** 427.31 429.4 |
| **Stroke/transient ischemic attack:** 435 435.1 435.2 435.3 435.8 435.9 433.81 433.91 434 436 437 437.1 433.31 433.01 434.01 434.1 434.11 434.9 434.91 437.2 437.3 437.4 437.5 437.6 437.7 437.8 437.9 430 431 432 432.1 432.9 |
| **Ischemic heart disease:** 410.01 410.02 410.1 410.11 410.12 410.2 410.21 410.22 410.3 410.31 410.32 410.4 410.41 410.42 410.5 410.51 410.52 410.6 410.61 410.62 410.7 410.71 410.72 410.8 410.81 410.82 410.9 410.91 410.92 411 411.1 411.8 411.81 411.89 413 413.1 413.9 414 414.01 414.02 414.03 414.04 414.05 414.06 414.07 414.1 414.11 414.12 414.19 414.2 414.3 414.4 414.8 414.9 410 412 |
| **Peripheral vascular disease:** 250.7 443.9 443 443.1 443.2 443.21 443.22 443.23 443.24 443.29 443.8 443.81 443.82 443.89 441 443.9 785.4 V43.4 |
| **Acute myocardial infarction:** 410 410.01 410.02 410.1 410.11 410.12 410.2 410.21 410.22 410.3 410.31 410.32 410.4 410.41 410.42 410.5 410.51 410.52 410.6 410.61 410.62 410.7 410.71 410.72 410.8 410.81 410.82 410.9 410.91 410.92 |
| **H. pylori infection:** 041.86 (ICD-9 codes) + MIS data + H. pylori eradication therapy |
| **Renal diseases:** 582 582.1 582.2 582.4 582.8 582.81 582.89 582.9 583 583 583.1 583.2 583.4 583.6 583.7 585 585.1 585.2 585.3 585.4 585.5 585.6 585.9 586 588 588 588.1 588.8 588.81 588.89 588.9 |
| **Venous thromboembolism:** 415.1 451, 453.1 453.2 453.3 453.8 453.9 |

# Supplementary Table 2. Calculations for variability measure

| **Variability measure** | **Definition** |
| --- | --- |
| Standard deviation (SD) | $\sqrt{\frac{\sum_{i=1}^{Number of measurements} {({test}_{i}-individual mean)}^{2}}{Number of measurements}}$ |
| Variance | $\frac{\sum_{i=1}^{Number of measurements} {({test}_{i}-individual mean)}^{2}}{Number of measurements}$ |
| Standard deviation/Initial (SD/initial) | $\frac{SD}{individual initial value}$ |
| Coefficient of variation (CV) | $\frac{SD}{individual mean}$ |

.

# Supplementary Table 3. Multivariable Cox regression models with adjustments to predict new gastric cancer and gastric diseases in the SGLT2I v.s. DPP4I matched cohort.

HR: hazard ratio; CI: confidence interval; SGLT2I: sodium glucose cotransporter-2 inhibitor; DPP4I: dipeptidyl peptidase-4 inhibitor.

Model 1 adjusted for significant demographics.

Model 2 adjusted for significant demographics, and past comorbidities.

Model 3 adjusted for significant demographics, past comorbidities, and non-SGLT2I/DPP4I medications.

Model 4 adjusted for significant demographics, past comorbidities, non-SGLT2I/DPP4I medications, abbreviated MDRD, and NLR

Model 5 adjusted for significant demographics, past comorbidities, non-SGLT2I/DPP4I medications, abbreviated MDRD, NLR, fasting glucose, HbA1c, and duration from earliest diabetes mellitus date to initial drug exposure date.

| **Characteristics** | **Peptic ulcer**  **HR [95% CI];P value** | **Acute gastritis**  **HR [95% CI];P value** | **Non acute gastritis**  **HR [95% CI];P value** | **Gastroesophageal reflux disease**  **HR [95% CI];P value** | **Gastric cancer**  **HR [95% CI];P value** | **All-cause mortality**  **HR [95% CI];P value** |
| --- | --- | --- | --- | --- | --- | --- |
| ***Model 1*** | 0.50[0.39-0.63];<0.0001 | 0.33[0.25-0.45];<0.0001 | 0.33[0.25-0.42];<0.0001 | 0.73[0.64-0.83];<0.0001 | 0.27[0.19-0.38];<0.0001 | 0.56[0.51-0.62];<0.0001 |
| ***Model 2*** | 0.49[0.38-0.62];<0.0001 | 0.33[0.25-0.44];<0.0001 | 0.32[0.25-0.41];<0.0001 | 0.71[0.62-0.81];<0.0001 | 0.26[0.18-0.37];<0.0001 | 0.55[0.50-0.60];<0.0001 |
| ***Model 3*** | 0.534[0.419-0.680];<0.0001 | 0.387[0.288-0.520];<0.0001 | 0.342[0.264-0.443];<0.0001 | 0.798[0.693-0.918];0.0016 | 0.277[0.195-0.392];<0.0001 | 0.63[0.57-0.69];<0.0001 |
| ***Model 4*** | 0.58[0.43-0.78];0.0003 | 0.30[0.20-0.44];<0.0001 | 0.32[0.23-0.43];<0.0001 | 0.60[0.50-0.72];<0.0001 | 0.27[0.18-0.41];<0.0001 | 0.73[0.64-0.83];<0.0001 |
| ***Model 5*** | 0.66[0.47-0.91];0.0118 | 0.31[0.20-0.48];<0.0001 | 0.35[0.25-0.49];<0.0001 | 0.62[0.50-0.76];<0.0001 | 0.30[0.19-0.48];<0.0001 | 0.77[0.66-0.89];0.0003 |

# Supplementary Table 4. Sensitivity analysis: Three-arm (only SGLT2I, only DPP4I, and only GLP1a) analysis results using stabilized IPTW.

The following exclusion criteria applied

1. With prior peptic ulcer, gastritis, gastroesophageal reflux disease, and gastric cancer (N=108)

2. Patients who died within 30 days after initial drug exposure (N=167)

3. Without complete demographics (N=19)

4. Under 18 years old (N=108)

5. Used at least two drugs from SGLT21, DPP4I, and GLP1a (N=12956)

6. New onset gastric cancer development less than 1 year after drug exposure (N=29)

HR: hazard ratio; CI: confidence interval; SGLT2I: sodium glucose cotransporter-2 inhibitor; DPP4I: dipeptidyl peptidase-4 inhibitor.

| **Model** | **Peptic ulcer**  **HR [95% CI];P value** | **Acute gastritis**  **HR [95% CI];P value** | **Non acute gastritis**  **HR [95% CI];P value** | **Gastroesophageal reflux disease**  **HR [95% CI];P value** | **Gastric cancer**  **HR [95% CI];P value** | **All-cause mortality**  **HR [95% CI];P value** |
| --- | --- | --- | --- | --- | --- | --- |
| DPP4I v.s. SGLT2I | 2.19[1.76-2.72];<0.0001 | 3.06[2.33-4.01];<0.0001 | 2.25[1.89-2.68];<0.0001 | 1.58[1.40-1.78];<0.0001 | 2.18[1.69-2.80];<0.0001 | 2.57[2.36-2.79];<0.0001 |
| GLP1a v.s. SGLT2I | 1.57[0.80-3.05];=0.1864 | 1.03[0.49-2.19];=0.9343 | 1.51[0.99-2.32];=0.5818 | 1.43[1.04-1.97];=0.0299 | 2.47[1.27-4.81];=0.0079 | 0.84[0.61-1.15];= 0.2659 |

# Supplementary Table 5. Sensitivity analyses: Exposure effects of SGLT2I v.s. DPP4I on new onset gastric diseases in the SGLT2I v.s. DPP4I matched cohort across different hazard models and matching methods.

SGLT2I: Sodium-glucose cotransporter-2 inhibitors; DPP4I: Dipeptidyl peptidase-4 inhibitors; HR: hazard ratio; CI: confidence interval

| **Model** | **Peptic ulcer**  **HR [95% CI];P value** | **Acute gastritis**  **HR [95% CI];P value** | **Non acute gastritis**  **HR [95% CI];P value** | **Gastroesophageal reflux disease**  **HR [95% CI];P value** | **Gastric cancer**  **HR [95% CI];P value** | **All-cause mortality**  **HR [95% CI];P value** |
| --- | --- | --- | --- | --- | --- | --- |
| Cause-specific hazard models | 0.52[0.41-0.67];<0.0001 | 0.354[0.265-0.474];<0.0001 | 0.348[0.270-0.449];<0.0001 | 0.756[0.659-0.867];0.0001 | 0.282[0.199-0.398];<0.0001 | 0.60[0.55-0.66];<0.0001 |
| Sub-distribution hazard models | 0.49[0.38-0.62];<0.0001 | 0.33[0.24-0.44];<0.0001 | 0.32[0.25-0.41];<0.0001 | 0.71[0.62-0.82];<0.0001 | 0.26[0.19-0.37];<0.0001 | 0.55[0.50-0.60];<0.0001 |
| PS stratification | 0.66[0.47-0.91];0.0124 | 0.31[0.20-0.49];<0.0001 | 0.34[0.24-0.48];<0.0001 | 0.62[0.50-0.76];<0.0001 | 0.30[0.19-0.47];<0.0001 | 0.77[0.66-0.89];0.0003 |
| PS with IPTW | 0.52[0.40-0.67];<0.0001 | 0.35[0.25-0.48];<0.0001 | 0.33[0.26-0.44];<0.0001 | 0.67[0.57-0.78];<0.0001 | 0.27[0.19-0.39];<0.0001 | 0.64[0.58-0.72];<0.0001 |
| PS with SIPTW | 0.48[0.38-0.61];<0.0001 | 0.33[0.24-0.43];<0.0001 | 0.32[0.25-0.41];<0.0001 | 0.71[0.62-0.81];<0.0001 | 0.26[0.18-0.37];<0.0001 | 0.55[0.50-0.60];<0.0001 |

# Supplementary Table 6. Sensitivity analysis: Excluding patients with CKD stage 4/5 (eGFR <30), peritoneal dialysis or haemodialysis in the SGLT2I v.s. DPP4I matched cohort.

SGLT2I: Sodium-glucose cotransporter-2 inhibitors; DPP4I: Dipeptidyl peptidase-4 inhibitors; HR: hazard ratio; CI: confidence interval

|  | **Peptic ulcer HR [95% CI];P value** | **Acute gastritis HR [95% CI];P value** | **Non-acute gastritis HR [95% CI];P value** | **Gastroesophageal reflux disease HR [95% CI];P value** | **Gastric cancer HR [95% CI];P value** | **All-cause mortality HR [95% CI];P value** |
| --- | --- | --- | --- | --- | --- | --- |
| SGLT2I v.s. DPP4I | 0.45[0.35-0.58];<0.0001 | 0.29[0.21-0.40];<0.0001 | 0.31[0.24-0.40];<0.0001 | 0.59[0.51-0.69];<0.0001 | 0.25[0.18-0.36];<0.0001 | 0.55[0.50-0.61];<0.0001 |

# Supplementary Table 7. Sensitivity analysis: Consideration of 1-year lag time effects in the SGLT2I v.s. DPP4I matched cohort.

SGLT2I: Sodium-glucose cotransporter-2 inhibitors; DPP4I: Dipeptidyl peptidase-4 inhibitors; HR: hazard ratio; CI: confidence interval

|  | **Peptic ulcer**  **HR [95% CI];P value** | **Acute gastritis**  **HR [95% CI];P value** | **Non-acute gastritis**  **HR [95% CI];P value** | **Gastroesophageal reflux disease**  **HR [95% CI];P value** | **Gastric cancer**  **HR [95% CI];P value** | **All-cause mortality**  **HR [95% CI];P value** |
| --- | --- | --- | --- | --- | --- | --- |
| SGLT2I v.s. DPP4I | 0.36[0.27-0.48];<0.0001 | 0.33[0.23-0.46];<0.0001 | 0.35[0.26-0.47];<0.0001 | 0.79[0.67-0.92];0.0028 | 0.26[0.18-0.37];<0.0001 | 0.55[0.50-0.61];<0.0001 |

# Supplementary Table 8. Sensitivity analysis: As-treated approach of SGLT2I v.s. DPP4I on new onset gastric diseases in the SGLT2I v.s. DPP4I matched cohort

SGLT2I: Sodium-glucose cotransporter-2 inhibitors; DPP4I: Dipeptidyl peptidase-4 inhibitors; HR: hazard ratio; CI: confidence interval

|  | **Peptic ulcer**  **HR [95% CI];P value** | **Acute gastritis**  **HR [95% CI];P value** | **Non-acute gastritis**  **HR [95% CI];P value** | **Gastroesophageal reflux disease**  **HR [95% CI];P value** | **Gastric cancer**  **HR [95% CI];P value** | **All-cause mortality**  **HR [95% CI];P value** |
| --- | --- | --- | --- | --- | --- | --- |
| SGLT2I v.s. DPP4I | 0.40[0.33-0.48];<0.0001 | 0.23[0.18-0.29];<0.0001 | 0.32[0.28-0.38];<0.0001 | 0.57[0.52-0.64];<0.0001 | 0.28[0.23-0.36];<0.0001 | 0.19[0.18-0.21];<0.0001 |

# Supplementary Table 9. Falsification analysis: Exposure effects of SGLT2I v.s. DPP4I on new onset venous thromboembolism in the SGLT2I v.s. DPP4I matched cohort after 1:1 propensity score matching

SGLT2I: Sodium-glucose cotransporter-2 inhibitors; DPP4I: Dipeptidyl peptidase-4 inhibitors; HR: hazard ratio; CI: confidence interval

Adjusted for significant demographics, past comorbidities, non-SGLT2I/DPP4I medications, abbreviated MDRD, NLR, fasting glucose, HbA1c, and duration from earliest diabetes mellitus date to initial drug exposure date.

|  | **Number of events, Count(%)** | **Time to venous thromboembolism, days** | **Adjusted hazard ratio, HR [95% CI]** | **P value** |
| --- | --- | --- | --- | --- |
| SGLT2I | 192(0.81%) | 2051.0(1949.0-2126.0) | 1.21[0.98-1.53] | 0.1034 |
| DPP4I | 159(0.67%) | 2042.0(1935.0-2122.0) | 1 [Reference] | N/A |

# Supplementary Table 10. The basic characteristics of the SGLT2I, DPP4I and GLP1a patients in the 3-arm analysis.

SGLT2I: sodium glucose cotransporter-2 inhibitor; DPP4I: dipeptidyl peptidase-4 inhibitor; SD: standard deviation; RMS: Rooted mean square; CV: Coefficient of variation; MDRD: modification of diet in renal disease; ACEI: angiotensin-converting enzyme inhibitors; ARB: angiotensin II receptor blockers; HBV: hepatitis B virus; HCV: hepatitis C virus; ^a^: N (%); ^b^: Median (IQR); ^c^: Mean (SD)

| **Characteristics** | **All**  **(N=46884)** | **DPP4I users (N=22052)** | **GLP1a users (N=2951)** | **SGLT2I users (N=21881)** | **P value** |
| --- | --- | --- | --- | --- | --- |
| ***Demographics*** |  |  |  |  |  |
| Female gender ^a^ | 18892 (40.3) | 8759 (39.7) | 1321 (44.8) | 8812 (40.3) | <0.001 |
| Male gender ^a^ | 27992 (59.7) | 13293 (60.3) | 1630 (55.2) | 13069 (59.7) |  |
| Baseline age, years ^b^ | 58.1 (13.6) | 58.7 (13.5) | 53.2 (12.8) | 58.3 (13.9) | <0.001 |
| ***Past Comorbidities*** |  |  |  |  |  |
| Charlson comorbidity index ^b^ | 1.0 (1.0) | 1.0 (1.0) | 1.0 (2.0) | 1.0 (1.0) | <0.001 |
| Duration from earliest diabetes mellitus diagnosis date-baseline date, day ^b^ | 6.1 (8.8) | 5.5 (8.8) | 6.0 (8.5) | 6.7 (8.7) | <0.001 |
| Hypertension ^c^ | 10149 (21.6) | 4641 (21.0) | 659 (22.3) | 4849 (22.2) | 0.012 |
| Heart failure ^c^ | 1242 (2.6) | 582 (2.6) | 71 (2.4) | 589 (2.7) | 0.657 |
| Ischemic heart disease ^c^ | 5575 (11.9) | 2586 (11.7) | 229 (7.8) | 2760 (12.6) | <0.001 |
| Atrial fibrillation ^c^ | 1045 (2.2) | 515 (2.3) | 20 (0.7) | 510 (2.3) | <0.001 |
| Acute myocardial infarction ^c^ | 1565 (3.3) | 748 (3.4) | 57 (1.9) | 760 (3.5) | <0.001 |
| Peripheral vascular disease ^c^ | 384 (0.8) | 185 (0.8) | 18 (0.6) | 181 (0.8) | 0.425 |
| Renal diseases ^c^ | 301 (0.6) | 144 (0.7) | 16 (0.5) | 141 (0.6) | 0.777 |
| Stroke/transient ischemic attack ^c^ | 1221 (2.6) | 590 (2.7) | 50 (1.7) | 581 (2.7) | 0.006 |
| Gastrointestinal bleeding ^c^ | 815 (1.7) | 389 (1.8) | 35 (1.2) | 391 (1.8) | 0.059 |
| H. pylori infection ^c^ | 2388 (5.1) | 1104 (5.0) | 146 (4.9) | 1138 (5.2) | 0.607 |
| Autoimmune disease tissue ^c^ | 480 (1.0) | 222 (1.0) | 31 (1.1) | 227 (1.0) | 0.94 |
| Cancer ^c^ | 894 (1.9) | 433 (2.0) | 37 (1.3) | 424 (1.9) | 0.027 |
| Chronic liver disease and cirrhosis ^c^ | 1245 (2.7) | 574 (2.6) | 103 (3.5) | 568 (2.6) | 0.014 |
| HBV infection ^c^ | 2365 (5.0) | 1083 (4.9) | 126 (4.3) | 1156 (5.3) | 0.028 |
| HCV infection ^c^ | 140 (0.3) | 68 (0.3) | 6 (0.2) | 66 (0.3) | 0.613 |
| Other liver diseases ^c^ | 441 (0.9) | 215 (1.0) | 20 (0.7) | 206 (0.9) | 0.291 |
| Diabetic retinopathy ^c^ | 3038 (6.5) | 1309 (5.9) | 261 (8.8) | 1468 (6.7) | <0.001 |
| ***Medication Prescriptions*** |  |  |  |  |  |
| Metformin ^c^ | 43866 (93.6) | 20603 (93.4) | 2909 (98.6) | 20354 (93.0) | <0.001 |
| Sulphonylurea ^c^ | 34646 (73.9) | 16861 (76.5) | 1795 (60.8) | 15990 (73.1) | <0.001 |
| Insulin ^c^ | 24030 (51.3) | 10931 (49.6) | 2407 (81.6) | 10692 (48.9) | <0.001 |
| Acarbose ^c^ | 1581 (3.4) | 666 (3.0) | 169 (5.7) | 746 (3.4) | <0.001 |
| Thiozolidinedone ^c^ | 12007 (25.6) | 4922 (22.3) | 1447 (49.0) | 5638 (25.8) | <0.001 |
| H2 antagonists ^c^ | 21747 (46.4) | 10297 (46.7) | 1392 (47.2) | 10058 (46.0) | 0.21 |
| Proton pump inhibitors ^c^ | 13720 (29.3) | 6128 (27.8) | 1284 (43.5) | 6308 (28.8) | <0.001 |
| ACEI/ARB ^c^ | 20270 (43.2) | 9427 (42.7) | 1525 (51.7) | 9318 (42.6) | <0.001 |
| Antibiotics ^c^ | 30127 (64.3) | 14260 (64.7) | 2049 (69.4) | 13818 (63.2) | <0.001 |
| Antihypertensive drugs ^c^ | 2688 (5.7) | 1113 (5.0) | 174 (5.9) | 1401 (6.4) | <0.001 |
| Antihepatitis ^c^ | 923 (2.0) | 444 (2.0) | 37 (1.3) | 442 (2.0) | 0.015 |
| Statins and fibrates ^c^ | 32519 (69.4) | 14798 (67.1) | 2219 (75.2) | 15502 (70.8) | <0.001 |
| HCV treatment drugs ^c^ | 723 (1.5) | 348 (1.6) | 29 (1.0) | 346 (1.6) | 0.039 |
| HBV treatment drugs ^c^ | 878 (1.9) | 425 (1.9) | 32 (1.1) | 421 (1.9) | 0.005 |
| Antiplatelets ^c^ | 11382 (24.3) | 5312 (24.1) | 577 (19.6) | 5493 (25.1) | <0.001 |
| Lipid-lowering drugs ^c^ | 20875 (44.5) | 9879 (44.8) | 1343 (45.5) | 9653 (44.1) | 0.191 |
| Non-steroidal anti-inflammatory drugs ^c^ | 10721 (22.9) | 5068 (23.0) | 542 (18.4) | 5111 (23.4) | <0.001 |
| Diuretics ^c^ | 11021 (23.5) | 5101 (23.1) | 744 (25.2) | 5176 (23.7) | 0.034 |
| Beta-blockers ^c^ | 9052 (19.3) | 4234 (19.2) | 545 (18.5) | 4273 (19.5) | 0.336 |
| Calcium channel blockers ^c^ | 15982 (34.1) | 7535 (34.2) | 1134 (38.4) | 7313 (33.4) | <0.001 |
| ***Liver and Renal Functions*** |  |  |  |  |  |
| Urea, mmol/L ^b^ | 5.4 (2.2) | 5.5 (2.3) | 5.3 (2.1) | 5.4 (2.2) | <0.001 |
| Creatinine, μmol/L ^b^ | 76.0 (27.0) | 77.0 (28.0) | 73.0 (27.1) | 75.0 (26.3) | <0.001 |
| Bilirubin, μmol/L ^b^ | 10.4 (5.8) | 10.3 (6.0) | 10.0 (5.7) | 10.6 (6.0) | <0.001 |
| ***Lipid/Glucose profiles*** |  |  |  |  |  |
| Triglyceride, mmol/L ^b^ | 1.4 (1.1) | 1.4 (1.1) | 1.7 (1.3) | 1.4 (1.1) | <0.001 |
| Low-density lipoprotein, mmol/L ^b^ | 2.3 (1.0) | 2.3 (1.0) | 2.3 (0.9) | 2.3 (1.0) | 0.402 |
| High-density lipoprotein, mmol/L ^b^ | 1.1 (0.3) | 1.1 (0.4) | 1.1 (0.4) | 1.1 (0.3) | <0.001 |
| Total cholesterol, mmol/L ^b^ | 4.2 (1.2) | 4.2 (1.2) | 4.3 (1.0) | 4.2 (1.2) | <0.001 |
| Baseline-Fasting glucose, mmol/L ^b^ | 8.4 (3.5) | 8.4 (3.2) | 9.6 (4.8) | 8.4 (3.6) | <0.001 |
| Baseline-Haemoglobin A1C, % ^b^ | 8.0 (1.8) | 7.9 (1.6) | 8.7 (2.0) | 7.9 (1.8) | <0.001 |
